# Integrated targeted sequencing reveals unique tissue-of-origin and donor-derived cell-free DNA signatures in stable organ transplant recipients

**DOI:** 10.1016/j.isci.2026.115315

**Published:** 2026-03-30

**Authors:** Nicholas Kueng, Fanny Sandberg, Daniel Sidler, Vanessa Banz, Annalisa Berzigotti, Charlotte K.Y. Ng, Carlo R. Largiadèr, Ursula Amstutz

**Affiliations:** 1 Department of Clinical Chemistry, Inselspital, Bern University Hospital, University of Bern, Bern, Switzerland; 2 Graduate School for Cellular and Biomedical Sciences, University of Bern, Bern, Switzerland; 3 Department of Nephrology and Hypertension, Inselspital, Bern University Hospital, University of Bern, Bern, Switzerland; 4 Department of Visceral Surgery and Medicine, Inselspital, Bern University Hospital, University of Bern, Bern, Switzerland; 5 Department of Hepatology, Inselspital, Bern University Hospital, University of Bern, Bern, Switzerland; 6 IRCCS Humanitas Research Hospital, Milan, Italy; 7 Humanitas University, Milan, Italy

**Keywords:** Biological sciences, Immunology, precision medicine

## Abstract

Solid organ transplantation is a life-saving treatment for end-stage organ disease, yet current methods to assess graft health rely on invasive biopsies. We applied an integrated targeted deep sequencing assay to simultaneously quantify donor-derived and tissue-of-origin (TOO) cell-free DNA (cfDNA) in plasma from liver and kidney transplant recipients and healthy individuals. The assay sensitively detected cell-type-specific cfDNA and revealed that cfDNA composition differed markedly between stable transplant recipients and healthy individuals. Early after transplantation, dynamic cfDNA changes were observed reflecting tissue-specific injury and recovery processes unique to each organ type. These findings show the potential of combining donor-derived and TOO cfDNA analysis to provide a more comprehensive view of transplant recipient health and possibly improve non-invasive monitoring of both graft-related and systemic complications.

## Highlights

• Single assay measuring both donor-derived cell-free DNA (cfDNA) and cfDNA tissue-of-origin
• Higher cfDNA from multiple non-graft tissues in stable liver and kidney recipients
• Increased cfDNA from 11 (kidney) and 25 (liver) cell types vs. healthy controls
• Early donor-derived cfDNA may include non-graft immune sources

## Graphical abstract

**Figure:**
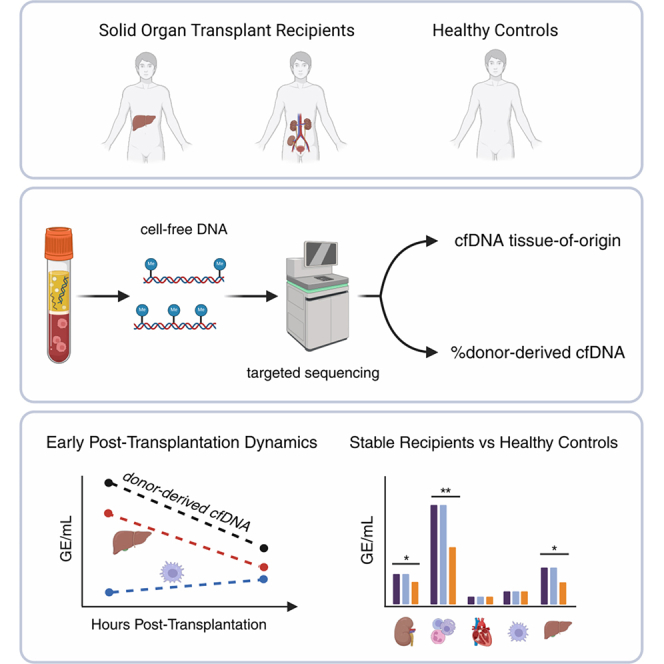

## Introduction

Organ transplantation is often the best treatment for end-stage organ disease. Despite improvements in survival rates and quality of life for patients, it requires life-long immunosuppressive therapy to prevent the rejection of the transplanted organ. Calcineurin inhibitors (CNIs) are frequently employed as a basis of immunosuppressive regimens because of their effectiveness in preventing cellular rejection. However, long-term CNI use has been linked to complications such as chronic kidney injury, metabolic disorders, cardiovascular events, and an elevated risk of malignancies.^1^ While managing these risks is critical, accurately monitoring allograft health is equally essential. Invasive tissue biopsy remains the gold standard for diagnosing allograft rejection and distinguishing it from other forms of graft injury, yet it carries inherent risks and places a significant burden on patients and the healthcare system, particularly when used in a surveillance setting.

Donor-derived cell-free DNA (dd-cfDNA) is emerging as a powerful, non-invasive biomarker that reflects tissue injury and cellular damage, making it a promising tool for allograft monitoring^2^^,^^3^^,^^4^ with the potential to reduce the need for tissue biopsies.^5^ Current methods for dd-cfDNA quantification, such as next-generation sequencing (NGS) and droplet digital PCR (ddPCR), leverage genetic differences between donor and recipient by analyzing single-nucleotide polymorphisms (SNPs),^6^^,^^7^ indels,^8^ or mismatches in the *HLA-DRB1* gene.^9^ However, dd-cfDNA methods have limitations and cannot be applied effectively in all clinical scenarios, such as when the donor and recipient are identical twins or when the recipient has received multiple organs from the same donor. Moreover, changes in total cfDNA due to inflammatory illness or leukopenia can alter the dd-cfDNA fraction and lead to false-positive or false-negative results, whereas absolute dd-cfDNA quantification is not affected.^10^ Additionally, some dd-cfDNA may not originate from the transplanted organ itself but from co-transplanted tissue or donor-derived immune cells, particularly in the early post-transplantation period.^11^

An alternative approach to detect cfDNA from the transplanted organ, without relying on donor-recipient genetic differences, involves tissue-of-origin (TOO)-specific DNA methylation patterns. DNA methylation is an epigenetic modification that is distinct for every cell type and is stable and highly reproducible across individuals,^12^ enabling the identification of cfDNA derived from specific cell types, including the transplanted organ. By examining unique methylation signatures associated with individual cell types, this approach has shown the potential to accurately determine the source of cfDNA.^11^^,^^12^^,^^13^^,^^14^ This strategy could not only be particularly useful in cases where genotype-based dd-cfDNA detection is ineffective but also expand our understanding of cfDNA release in transplant recipients beyond the allograft. However, current methylation-based approaches face technical challenges. Whole-genome bisulfite sequencing (WGBS) provides comprehensive CpG coverage but is too costly for routine clinical use, mainly when performed at higher depths to detect cell types of lower abundance. Widely used targeted methods, such as arrays or reduced-representation bisulfite sequencing, miss key CpG sites informative of the TOO,^12^ limiting their utility in such studies. Targeted sequencing offers a balanced solution, enabling a focus on the most informative CpG sites at high sequencing depth to enhance resolution with the possibility of extending to other markers of interest while remaining cost-effective and suitable for high-throughput applications.

While previous studies have investigated the dynamics of dd-cfDNA in transplant recipients,^15^^,^^16^^,^^17^ an important knowledge gap remains regarding the precise origin of cfDNA in the critical early post-transplantation phase. Existing methods distinguishing cfDNA based on donor-recipient genetic differences are unable to identify the specific cellular or tissue sources contributing to cfDNA levels immediately after transplantation, thus possibly not fully leveraging the diagnostic potential. Furthermore, while dd-cfDNA levels in stable transplant recipients have been relatively well-characterized, it is still unclear how the release of cfDNA from a transplanted organ compares to that from healthy individuals or how cfDNA release from recipient tissues, possibly influenced by immunosuppressive therapy or immunologic processes related to the allograft, may be altered in the overall cfDNA pool. Increasing the knowledge of such baseline data can thus improve the use of cfDNA as a marker for transplant health by increasing our understanding of cfDNA release from donor and recipient tissues, which may vary between organ type or recipient condition.

Herein, we describe a newly designed targeted deep sequencing assay leveraging multi-omic information to simultaneously and accurately determine both cfDNA TOO and dd-cfDNA proportions and quantities. We applied this assay in different phases of the solid organ transplant journey, including early after transplantation and through the first year. This approach enabled us to investigate cfDNA release dynamics and to further compare cfDNA profiles between stable transplant recipients and healthy individuals, as well as between kidney and liver recipients.

## Results

### Experimental approach

**Figure 1:**
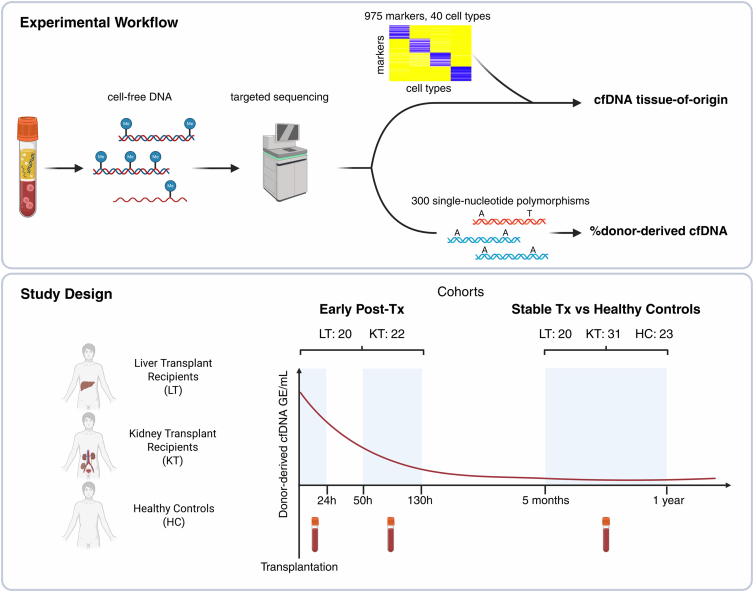
Study overview with experimental workflow and study design cfDNA was extracted from blood samples. Enzymatically converted cfDNA libraries were enriched for TOO informative markers and SNPs for dd-cfDNA quantification using a novel hybridization capture panel. The bioinformatic analysis resulted in two key metrics: cfDNA TOO after deconvolution based on 975 markers across 40 cell types and dd-cfDNA quantification using 300 SNPs. In the early post-Tx cohort, blood was collected twice per patient early post-transplantation: the first sample within 24 h and the second between 50 and 130 h after transplantation. In the stable Tx vs. healthy control cohort, blood was collected from healthy control individuals and stable liver and kidney transplant recipients between 5 months and 1-year post-transplantation.

We developed an experimental workflow to analyze cfDNA in transplant recipients using targeted deep methylation sequencing. In this workflow, cfDNA extracted from plasma underwent enzymatic conversion, followed by single-stranded library preparation and hybridization capture for targeted enrichment using a custom panel. Targeted sequencing was then performed, enabling simultaneous determination of tissue-specific cfDNA methylation patterns, using a panel comprising 975 markers representative of 40 distinct cell types, and quantification of dd-cfDNA, based on 300 SNPs across the human genome (Figure 1). Fractional quantities obtained with this sequencing panel were combined with the fluorescence-based quantification of total cfDNA to derive absolute quantities (GE/mL). The method was then applied to plasma samples from stable liver (stableLT) and kidney transplant (stableKT) recipients, and profiles were compared to those from age- and sex-matched healthy controls (HCs, Figure 1). Finally, paired blood samples from liver transplant (LT) and kidney transplant (KT) recipients collected at two early post-transplant time points were examined to characterize the kinetics of cfDNA release and clearance (Figure 1).

### Validation of targeted sequencing assay for cfDNA tissue-of-origin and dd-cfDNA quantification

**Figure 2:**
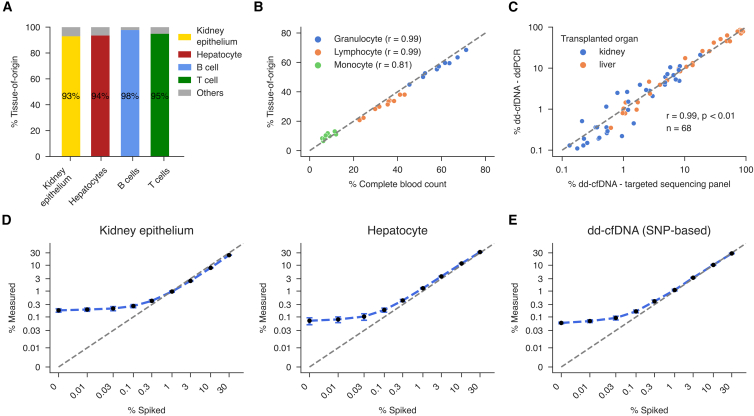
Analytical performance of the targeted sequencing workflow for cfDNA TOO determination and dd-cfDNA quantification (A) Stacked bar chart shows measured TOO of genomic DNA (gDNA) from primary human cell types: kidney epithelium (*n* = 1), hepatocytes (*n* = 1), B cells (*n* = 2), and T cells (*n* = 1). Percentages in each bar represent the proportion of the measured cell type. All non-target cell type proportions were summed up as “others”. (B) Correlation between measured TOO of whole blood gDNA and the corresponding granulocyte, lymphocyte, and monocyte complete blood count proportions from 10 HC. Each data series (colored points) represents one cell type with significant (*p* < 0.05) Pearson’s correlation coefficients shown in the legend. (C) Comparison of dd-cfDNA measurements by ddPCR (y axis) versus the targeted sequencing panel (x axis) for kidney (orange) and liver (blue) transplant recipients, demonstrating high concordance (Pearson’s r). (D) TOO deconvolution results of in silico mixtures. Reads from sequenced gDNA from kidney epithelium or hepatocyte samples were computationally mixed into a background of leukocyte gDNA reads from three HC. The background for the kidney epithelium mixtures consisted of 97% leukocyte and 3% hepatocyte reads. The mixture shown here had a sequencing depth of 1,000×. Black markers show the median-determined contribution for 20 replicates, with the error bars displaying one standard deviation. (E) dd-cfDNA proportion results of in silico mixtures. Plasma cfDNA reads from two HC were mixed at various fractions. The mixture shown here had a sequencing depth of 1,000×. Black markers show the median-determined contribution for 20 replicates, with the error bars displaying one standard deviation. The gray line represents the identity line, where displayed (y = x).

To validate our targeted sequencing panel, we analyzed pure primary human cell types relevant for transplant monitoring, including kidney epithelial cells, hepatocytes, B cells, and T lymphocytes. TOO analysis of these samples yielded 93 to 98% purity for the expected cell type, demonstrating the specificity of the panel (Figure 2A). Additionally, whole blood from HC (*n* = 10) was analyzed, revealing that the estimated cfDNA TOO proportions of granulocytes, monocytes, and lymphocytes were highly consistent with differential complete blood count (CBC) measurements (Pearson’s r = 0.81–0.99, *p* < 0.01; Figure 2B). In a subset of 68 transplant recipient samples, we independently quantified the fraction of dd-cfDNA (%dd-cfDNA) using a ddPCR-based method.^18^ The %dd-cfDNA determined by our targeted sequencing assay exhibited a strong correlation with quantities obtained by ddPCR^18^ (Pearson’s r = 0.99, *p* < 0.01; Figure 2C). We further assessed the workflow performance by conducting in silico mixture experiments. Reads from kidney epithelial cells or hepatocytes were computationally mixed with white blood cell (WBC) data from three HC (with an additional 3% hepatocytes included in kidney-WBC mixtures for a plasma-like background) at varying proportions and sequencing depths (20 replicates per combination). The workflow accurately detected kidney epithelial and hepatocyte fractions as low as 0.3% (Figure 2D), with the coefficient of variation (CV) decreasing with increasing sequencing depth. CVs below 20% were achieved at a depth of coverage of 400× or higher for spike-in fractions of 0.3% (Figure S1). A similar in silico approach was applied to dd-cfDNA quantification using mixtures of plasma cfDNA data from two HC. The workflow reliably detected dd-cfDNA at fractions as low as 0.1% (Figure 2E), with CVs below 20% at ≥400× coverage and below 30% at ≥300× coverage (Figure S2). Based on these in silico mixtures, linearity was confirmed for both TOO and dd-cfDNA quantification (Figures S3 and S4). In HC (*n* = 23), background levels were low, with a median of 0.097% (interquartile range, IQR: 0.073–0.136%) of non-HC allele signal. Across all plasma samples analyzed in this study (*n* = 158), the median sequencing coverage was 1,119× (IQR: 913–1,377) for the TOO informative regions and 884× (IQR: 745–1,082) for SNP targets. These depths are approximately 3-fold and 2-fold higher, respectively, than the minimum coverage required to achieve a CV equal to or smaller than 20% for detecting 0.3% cell type contributions (kidney epithelium and hepatocyte) and 0.1% dd-cfDNA. Lastly, the enzymatic conversion yielded good conversion rates with a median of 99.7% (IQR: 99.6–99.8%), with all samples exceeding 96%. Together, these results underscore the robustness and sensitivity of our workflow for both TOO analysis and dd-cfDNA quantification.

### TOO profiling reveals distinct cfDNA signatures in stable transplant recipients

**Figure 3:**
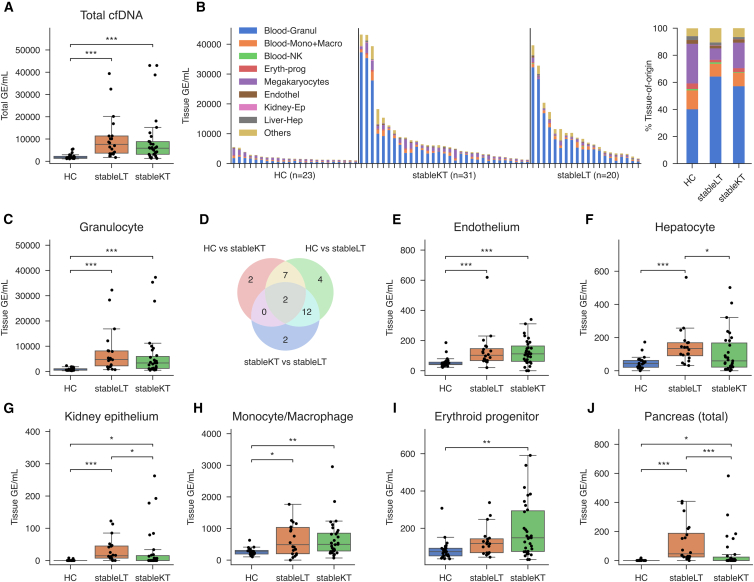
Comparative analysis of tissue-of-origin in healthy controls and stable transplant recipients (A) Total cfDNA. (B) Left: Absolute TOO GE/mL in individual samples, with bars grouped by cohort (HC, stableLT, stableKT) in descending order of total GE/mL. Right: Stacked bar chart of mean TOO proportions in HC, stableLT, and stableKT; Each colored segment represents a distinct cell type or group. (C) Boxplots comparing the absolute granulocyte cfDNA quantities across stableLT (*n* = 20) and stableKT (*n* = 31) recipients and HC (*n* = 23). (D) Venn diagram showing the number of cell types with significantly (*p* < 0.05*)* different cfDNA quantities across the three pairwise group comparisons. (E–J) Boxplots compare the absolute quantities originating from different cell types across the three groups: (E) endothelial cells, (F) hepatocytes, (G) kidney epithelium, (H) monocytes and macrophages, (I) erythroid progenitors, and (J) all cell types from the pancreas combined. The box spans the 25th to 75th percentiles with a line at the median. Whiskers extend to 1.5× the interquartile range. For each cell type, the Kruskal-Wallis test was performed for global comparisons, with the Benjamini-Hochberg procedure applied to control the false discovery rate (FDR) across cell types. For significant results, Dunn’s post-hoc test was used for pairwise comparisons between groups, and its *p* values were adjusted using the Bonferroni correction to account for multiple comparisons between groups. ∗*p* < 0.05, ∗∗*p* < 0.01, ∗∗∗*p* < 0.001, and ∗∗∗∗*p* < 0.0001, no comparison annotation means *p* > 0.05. See also Figures S6 and S8.

TOO deconvolution enables a detailed examination of cfDNA release in transplant recipients and potentially organ-specific effects of immunosuppressive therapy. To determine the cfDNA TOO signatures, total cfDNA was extracted and quantified from plasma of stableLT (*n* = 20) and stableKT (*n* = 31) recipients and compared with a sex- and age-matched HC cohort (*n* = 23, Table 1). Total plasma cfDNA was significantly elevated in transplant recipients (*p* < 0.001) with 4.5-fold higher levels in stableLT (genome equivalents/mL: GE/mL; median: 7,619 GE/mL, IQR: 3,644–11,370 GE/mL) and 3.5-fold higher levels in stableKT recipients (median: 5,878 GE/mL, IQR: 3,136–8,832 GE/mL) compared to HC (median: 1,627 GE/mL, IQR: 1,300–2,216 GE/mL; Figure 3A) with the majority (>95%; Figure S5) of the cfDNA being recipient-derived in both transplant recipient groups. To analyze the source of plasma cfDNA, we performed TOO deconvolution. Hematological-derived cfDNA constituted the largest fraction of total cfDNA in all three groups (Figures 3B and S6), with the relative contributions from different cell types varying considerably between groups, indicating distinct release dynamics in transplant recipients. To assess differences between groups for each cell type, the cell type-specific fractions were multiplied by the total concentration of cfDNA to calculate the absolute GE/mL. The overall higher cfDNA quantities in transplant recipients were predominantly driven by a significant increase in granulocyte-derived cfDNA (*p* < 0.0001; Figures 3B and 3C). Notably, transplant recipients also displayed a median of 3- to 5-fold higher levels of non-hematological cfDNA compared with HC (*p* < 0.001; Figure S7). As shown in Figure 3D, comparisons across 40 cell types among the three groups revealed differences in cfDNA quantities between at least two groups for 29 of the 40 cell types. Twelve cell types differed both between stableLT vs. HC and stableLT vs. stableKT, and seven differed between HC and both transplant groups. Only two cell types exhibited distinct differences between the two transplant groups, and for all significant comparisons, HC consistently displayed lower absolute cfDNA levels (Figure S8). Moreover, cfDNA from multiple cell types was uniquely detectable only in transplant patients. Among non-hematological cell types, endothelial cells and hepatocytes were the predominant contributors to cfDNA (Figures 3E and 3F). Given that nephrotoxicity mediated by endothelial injury is a well-recognized complication of CNIs,^19^ we examined tissue-specific cfDNA alterations for kidney epithelium. Most HC had no detectable cfDNA from the kidney epithelium, but unexpectedly, the stableLT group showed an increase with levels significantly higher than those in both the HC and the stableKT groups (Figure 3G). Similarly, endothelial-derived cfDNA was markedly increased (2-fold) in both transplant groups (*p* < 0.001; Figure 3E). In stableLT, hepatocyte-derived cfDNA was significantly higher with a 2-fold increase compared to HC and stableKT (Figure 3F). Interestingly, cfDNA from other major hematological sources, including monocytes/macrophages (*p* < 0.05; Figure 3H) and erythroid progenitors (*p* < 0.01; Figure 3I), also showed differences between the groups. In addition, cfDNA from all pancreatic cell types combined was elevated in transplant recipients compared to HC (*p* < 0.05; Figure 3J), with higher levels in stableLT compared to stableKT. Acinar, beta, and delta cell-derived cfDNA was increased exclusively in stableLT compared to HC (Figure S8).

**Table 1:** Demographic and clinical characteristics of the study population

Recipient Characteristics	Stable vs. Healthy Cohort			Early Post Transplantation Cohort	
	Healthy Controls (*n* = 23)	Stable Liver Recipients (*n* = 20)	Stable Kidney Recipients (*n* = 31)	Early post-TPL - Kidney (*n* = 22)	Early post-TPL - Liver (*n* = 20)
Sex, n (%)					
Male	18 (78.3%)	14 (70.0%)	20 (64.5%)	13 (59.1%)	12 (60.0%)
Female	5 (21.7%)	6 (30.0%)	11 (35.5%)	9 (40.9%)	8 (40.0%)
Age (yr), median [IQR]	54.0 [42.0, 58.0]	59.0 [47.3, 63.3]	54.0 [41.5, 65.5]	58.0 [43.3, 64.8]	61.0 [54.8, 63.5]
Time post-transplantation (days), median [IQR]	–	111.5 [79.5, 166.5]	101.0 [87.0, 174.0]	–	–
Immunosuppression medication, n (%)					
CNI + MPA/MMF + PRED	–	1 (5.0%)	29 (93.5%)	–	–
CNI + mTORi + MPA/MMF	–	2 (10.0%)	0 (0.0%)	–	–
CNI + MPA/MMF	–	10 (50.0%)	0 (0.0%)	–	–
CNI + PRED	–	2 (10.0%)	0 (0.0%)	–	–
CNI	–	4 (20.0%)	1 (3.2%)	–	–
mTORi + PRED	–	1 (5.0%)	0 (0.0%)	–	–
MPA/MMF + PRED	–	0 (0.0%)	1 (3.2%)	–	–
Total ischemia time^a^ (min.), median [IQR]	–	368 [319, 402]	327 [169, 402]	325 [185, 424]	399 [337, 447]
Donor type, n (%)					
Living	–	0 (0.0%)	14 (45.2%)	9 (40.9%)	0 (0.0%)
Deceased	–	20 (100.0%)	17 (54.8%)	13 (59.1%)	20 (100.0%)
Delayed graft function, n (%)					
Yes	–	–	–	7 (31.8%)	–
No	–	–	–	15 (68.2%)	–
Indication for transplantation, n (%)					
ADPKD	–	0 (0.0%)	6 (19.4%)	7 (31.8%)	1 (5.0%)
Diabetes/hypertension/vascular etiology	–	0 (0.0%)	6 (19.4%)	7 (31.8%)	0 (0.0%)
Glomerulosclerosis/-nephritis	–	0 (0.0%)	7 (22.6%)	3 (13.6%)	0 (0.0%)
Alcohol-related liver disease	–	3 (15.0%)	0 (0.0%)	0 (0.0%)	2 (10.0%)
Autoimmune	–	2 (10.0%)	0 (0.0%)	0 (0.0%)	2 (10.0%)
Hepatocellular carcinoma	–	6 (30.0%)	0 (0.0%)	0 (0.0%)	6 (30.0%)
Metabolic liver disease	–	3 (15.0%)	0 (0.0%)	0 (0.0%)	4 (20.0%)
Virus-induced liver cirrhosis	–	2 (10.0%)	0 (0.0%)	0 (0.0%)	2 (10.0%)
Other^b^	–	4 (20.0%)	12 (38.8%)	5 (22.7%)	3 (15.0%)

post-TPL, post-transplantation; IQR, interquartile range; CNI, calcineurin inhibitors (tacrolimus/cyclosporin A); mTORi, mTOR inhibitor (everolimus); MPA, mycophenolic acid; MMF, mycophenolate mofetil; PRED, prednisolone; ADPKD, autosomal dominant polycystic kidney disease.

a Cold and warm ischemia time combined.

b Includes COVID-19, genetic, trauma, birth defect, and so forth.

We also quantified dd-cfDNA, which is commonly reported as a fraction of the total cfDNA, and compared it to the proportion of graft-derived cfDNA determined through TOO deconvolution. In stableLT, the median %dd-cfDNA was 1.4% (IQR: 0.88–2.6%; Figure S5), which closely matched the median hepatocyte-derived cfDNA at 1.3% (IQR: 1.1–2.3%; *p* = 0.41). By contrast, stableKT samples exhibited a median %dd-cfDNA of 0.26% (IQR: 0.22–0.33%; Figure S5) and a median kidney epithelium contribution of 0.00% (IQR: 0.00–0.27%), a statistically significant difference (*p* < 0.001) given that most stableKT samples had kidney epithelium fractions below the estimated detection limit of 0.3%.

### Correlation analysis of cell type-specific cfDNA with clinical parameters in stable transplant recipients

To further elucidate the potential clinical significance of our cfDNA measurements, we assessed the correlations between cell-specific cfDNA levels and established biomarkers of immunosuppression, immune response, and organ injury. In a subset of stableKT (*n* = 20) with available matched cyclosporin A plasma levels, absolute endothelium-derived cfDNA levels correlated moderately with cyclosporin A concentrations (Pearson’s r = 0.54, *p* < 0.01; Figure S9), whereas kidney epithelial cfDNA (*p* = 0.91) or total cfDNA (*p* = 0.82) levels showed no correlation. Previous research suggests that immune cfDNA dynamics reflect immune response and turnover rather than changes in the peripheral cell counts.^20^ Here, matched immune cell concentrations in blood from the stable cohort revealed no correlation of total leukocyte counts with total cfDNA from leukocytes (*n* = 48; *p* = 0.94), total lymphocyte counts with total cfDNA from lymphocytes (*n* = 27; *p* = 0.60), monocyte counts with monocyte cfDNA (*n* = 28; *p* = 0.07), and total neutrophil counts with granulocyte cfDNA (*n* = 28; *p* = 0.30). Of note, the median leukocyte count was 5.8 × 10^9^/L (IQR: 4.1–8.3 × 10^9^/L), with most measurements (81%) within the reference range (3–10.5 × 10^9^/L). In a subset of patients with C-reactive protein (CRP) levels measured at the same time point, we did not observe any correlation with total cfDNA (*n* = 45; *p* = 0.61). Finally, liver enzyme measurements, which serve as established biomarkers for liver injury, were obtained for stableLT and stableKT recipients where available from the clinical records. Hepatocyte cfDNA showed a moderate correlation with gamma-glutamyl transferase (*n* = 35; Pearson’s r = 0.54, *p* < 0.01), alanine aminotransferase (ALAT; *n* = 39; Pearson’s r = 0.40, *p* < 0.05), and aspartate aminotransferase (ASAT; *n* = 33; Pearson’s r = 0.49, *p* < 0.01) (Figure S10). As HC were sex- and age-matched to stableLT and stableKT, no associations between sex or age with cell-specific cfDNA levels were investigated.

### Organ-specific dynamics of cfDNA release in the early post-transplant period

Early kinetics of dd-cfDNA have been shown to predict graft recovery and long-term function in KT recipients.^21^ However, the precise cellular origins of cfDNA during this critical early phase remain largely undefined. To address this, we evaluated the kinetics of cfDNA release from different cell types in LT and KT recipients by collecting blood samples at two time points. Blood samples were obtained from 20 LT and 22 KT recipients (Table 1) within 24 h post-transplant (median: 15 h, IQR: 12–20 h) and between 50 and 130 h post-transplantation (median: 69 h, IQR: 62–91 h). These intervals capture key phases of surgical recovery, including responses to trauma, ischemia-reperfusion injury, and tissue repair.

**Figure 4:**
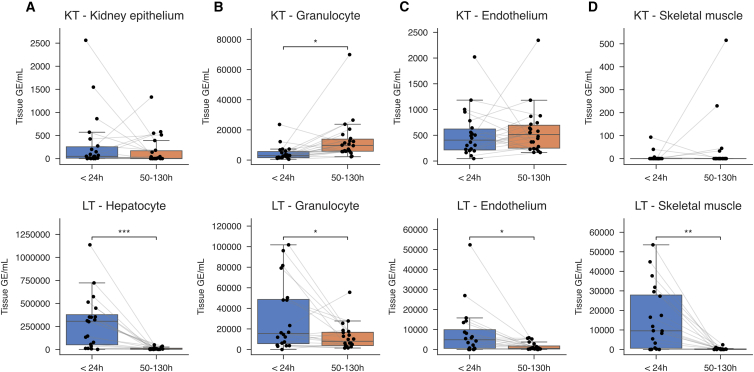
Differences in cell type-specific cfDNA release for kidney and liver transplant recipients early post-transplantation Cell type-specific cfDNA quantities in KT and LT recipients were measured at <24 h and 50–130 h post-transplantation. Shown are the cfDNA quantities from (A) the kidney epithelium in KT and hepatocytes in LT, (B) granulocytes from both, (C) endothelium from both, and (D) skeletal muscle from both, with gray lines connecting repeated measurements from the same individual. The Wilcoxon signed-rank test was used, and *p* values were adjusted for multiple comparisons across cell types using the Benjamini-Hochberg procedure to control the false discovery rate (FDR). ∗*p* < 0.05, ∗∗*p* < 0.01, ∗∗∗*p* < 0.001, and ∗∗∗∗*p* < 0.0001, no comparison annotation means *p* > 0.05. The box spans the 25th to 75th percentiles with a line at the median. Whiskers extend to 1.5× the interquartile range. See also Figures S12 and S13.

In KT recipients, total cfDNA levels were lower in the early post-transplantation period (median: 6,050 GE/mL, IQR: 4,357–12,845 GE/mL) and increased significantly at the later time point (median: 17,786 GE/mL, IQR: 12,953–32,374 GE/mL; *p* < 0.01; Figure S11A). Conversely, LT recipients exhibited substantially elevated cfDNA concentrations (median: 456,936 GE/mL, IQR: 143,150–693,807 GE/mL) that decreased sharply by 50–130 h (median: 27,396 GE/mL, IQR: 14,105–59,293 GE/mL; *p* < 0.001; Figure S11B). TOO deconvolution in LT recipients revealed that cfDNA from 22 of 40 cell types was significantly elevated in the early phase compared to the second time point (Figure S12). Interestingly, kidney epithelial cfDNA levels in KT recipients remained consistently low (*p* = 0.60; Figure 4A) for most patients. Conversely, LT recipients showed an almost 55-fold decrease in hepatocyte-derived cfDNA from the early to later phase (*p* < 0.001; Figure 4A), with granulocyte- and endothelium-derived cfDNA also decreasing (*p* < 0.05; Figures 4B and 4C). In KT recipients, significant changes between time points were primarily confined to granulocyte-derived cfDNA, which increased at 50–130 h (Figures 4B and S13). While skeletal muscle cfDNA was barely detectable in HC and KT early post-transplantation, LT recipients displayed significantly elevated skeletal muscle cfDNA levels within the first 24 h (median: 9,542 GE/mL, IQR: 645–27,876 GE/mL), which dropped markedly by 50–130 h post-transplantation (median: 132 GE/mL, IQR: 0–280 GE/mL; *p* < 0.01; Figure 4D). In KT recipients, neither endothelial nor skeletal muscle cfDNA levels differed significantly between the two time points (*p* > 0.05; Figures 4C and 4D).

Similar to the stable cohort, blood leukocyte cell counts were routinely measured in this cohort. However, we did not observe any significant correlation between matched total leukocyte counts and total leukocyte-derived cfDNA (*n* = 83; *p* = 0.64). This lack of correlation was consistent across each time point and transplanted organ (all correlations: *p* > 0.05). We also obtained liver enzyme measurements for this cohort, which were only available for LT recipients. We performed correlation analysis between these measurements and absolute hepatocyte cfDNA quantities. The analysis revealed significant correlations with ALAT within 24 h (*n* = 18; Pearson’s r = 0.57, *p* < 0.05) and 50–130 h post-transplantation (*n* = 20; Pearson’s r = 0.56, *p* < 0.01) (Figure S14). The findings were similar for ASAT within 24 h post-transplantation (*n* = 18; Pearson’s r = 0.80, *p* < 0.001), but the correlation was not significant 50–130 h post-transplantation (*n* = 19; *p* = 0.07) (Figure S14).

### Comparison of donor-derived and tissue-specific dynamics in post-transplant plasma

**Figure 5:**
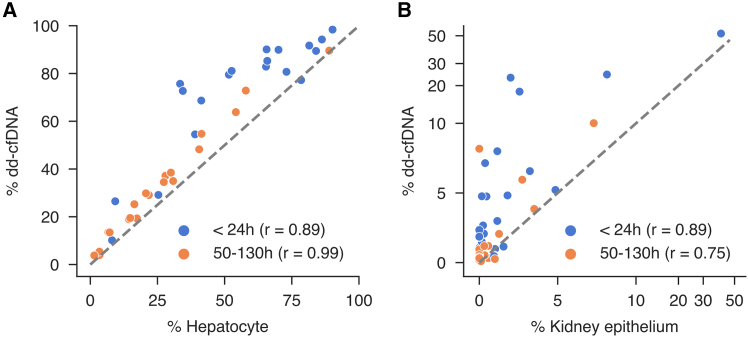
Correlation between plasma %dd-cfDNA and tissue-specific cfDNA fractions early post-transplantation (A) Correlation between plasma %dd-cfDNA and hepatocyte-derived cfDNA in LT recipients early post-transplant. (B) Correlation between %dd-cfDNA and kidney epithelium-derived cfDNA in KT recipients early post-transplant. The x- and y-axes each use a hybrid scale: values below 10% are displayed on a linear scale, while values above 10% are displayed on a log10 scale. For both plots, each color represents a different collection time point. Pearson’s correlation coefficients are shown (all with *p* < 0.05). The gray line represents the identity line (y = x).

Given that prior studies have predominantly focused on dd-cfDNA as a biomarker for allograft injury, the specific contribution of the primary transplanted tissue to the plasma cfDNA pool remains unclear. To elucidate this relationship, we quantified both the relative and absolute levels of dd-cfDNA in post-transplant samples using the integrated targeted sequencing method and compared these values to the graft-derived cfDNA determined by TOO deconvolution. In LT recipients, dd-cfDNA constituted 80.9% (IQR: 71.7–89.9%) of the total cfDNA in the early post-transplant period, decreasing to 29.5% (IQR: 17.6–40.9%) at the later time point. Similarly, absolute dd-cfDNA levels declined from 387,650 GE/mL (IQR: 76,238–562,589 GE/mL) to 7,666 GE/mL (IQR: 3,117–19,255 GE/mL; *p* < 0.001). Of note, %dd-cfDNA within 24 h post-transplantation was higher compared to hepatocyte-derived cfDNA in all patients (Figure 5A), with a median %dd-cfDNA not explained by hepatocyte-derived cfDNA of 17.3% (IQR: 8.0–25.2%). This non-hepatocyte %dd-cfDNA fraction correlated significantly with the granulocyte cfDNA proportion in the <24 h phase (Pearson’s r = 0.51, *p* < 0.01). In KT recipients, the percentage of dd-cfDNA decreased significantly from 3.9% (IQR: 1.6–7.0%) in samples collected within 24 h post-transplant to 0.53% (IQR: 0.38–1.7%) at 50–130 h (*p* < 0.001). In contrast, the absolute dd-cfDNA did not differ significantly between the two time points (median: 322 GE/mL, IQR: 140–847 GE/mL vs. median: 112 GE/mL, IQR: 57–360 GE/mL; *p* = 0.08). Notably, despite a median dd-cfDNA of 3.9% in the early phase, kidney epithelium accounted for only 0.98% (IQR: 0.18–2.0%) of total cfDNA. Several samples (*n* = 7) with dd-cfDNA in the range of 1.5–7% exhibited kidney epithelium contributions of less than 0.5% (Figure 5B), with a median difference of all samples between %dd-cfDNA and kidney epithelium-derived cfDNA of 2.4% (IQR: 1.1–6.2%). This non-kidney epithelium dd-cfDNA fraction correlated significantly with the endothelium cfDNA proportion at both time points (<24 h: Pearson’s r = 0.56, *p* = 0.01; 50–130 h: Pearson’s r = 0.64, *p* < 0.01). The discrepancy between the kidney epithelium cfDNA proportion and %dd-cfDNA was reduced in the later post-transplant period, where kidney epithelium-derived cfDNA more closely paralleled the overall %dd-cfDNA.

Within the post-transplant cohort, approximately one-third (7/22) of KT recipients experienced delayed graft function (DGF), defined by the need for hemodialysis within the first week post-transplant (Table 1). Although DGF is linked to damage in kidney epithelium and endothelium,^22^ we observed no significant differences in the absolute quantities of cfDNA derived from these cell types or in dd-cfDNA levels between patients with and without DGF, regardless of collection time point or the fold-change between the two time points. Similarly, no significant differences were observed when data were stratified by donor type or recipient sex. Also, no donor type- or recipient sex-specific differences were observed for hepatocyte- and endothelial-derived cfDNA in LT recipients. For all KT recipients, matched plasma creatinine measurements were available, except for one patient within 24 h post-transplantation. Correlation analysis between plasma creatinine and %dd-cfDNA in patients with and without DGF revealed no significant correlation (Figure S15).

## Discussion

This study demonstrates that our newly proposed targeted deep sequencing assay comprehensively evaluates cfDNA in transplant recipients by extending its analysis beyond traditional dd-cfDNA quantification. By incorporating TOO analysis, our multi-omics approach distinguishes the diverse cellular contributors to the circulating cfDNA pool in addition to quantifying dd-cfDNA, all while avoiding the need for resource-intensive whole-genome approaches. Whereas dd-cfDNA primarily reflects graft-derived injury, TOO analysis extends the assessment to cfDNA released from recipient tissues affected by systemic or treatment-related processes, potentially providing broader insight into complications relevant for transplant monitoring.

We observed that stable liver and kidney allograft recipients exhibited substantially higher total cfDNA levels than healthy individuals. This finding aligns with prior studies that reported similarly elevated cfDNA levels in long-term stable kidney^16^ and heart transplant^23^ patients, potentially reflecting continuous cell turnover and increased injury to recipient tissue beyond the allograft. Our integrated approach, with its ability to reveal tissue-specific cfDNA release patterns, provides insight into the source of this increased recipient cfDNA. For instance, we observed that granulocyte-derived cfDNA was strongly increased in both stableLT and stableKT recipients compared to HC and was the dominant source of cfDNA in the stable phase. Similarly, cfDNA composition, particularly in granulocyte-derived cfDNA, also showed different dynamics between KT and LT recipients in the early post-transplant period. The lack of correlations between immune cell counts and their corresponding cfDNA levels suggests that these dynamics in cfDNA release are driven by altered cell turnover, immune responses or surgical trauma rather than corresponding changes in circulating cell count.^20^ One of the primary origins of granulocyte cfDNA is likely the formation of neutrophil or eosinophil extracellular traps (NET, EET), consistent with previous work showing increased neutrophil-derived cfDNA as a surrogate marker for NETosis.^24^^,^^25^ The observed increase in granulocyte-derived cfDNA in stable transplant recipients may thus indicate ongoing neutrophil activity, including increased NET formation. Previous work has shown significantly increased levels of a marker for NETosis after transplantation in patients with previous end-stage renal disease.^26^ Excessive NET formation has also been linked to graft dysfunction and rejection in both kidney and LT recipients.^27^^,^^28^ The elevated cfDNA levels from granulocytes in stable recipients may suggest a state of controlled neutrophil activation that, while not triggering overt rejection, may subtly influence graft microenvironments and potentially contribute to subclinical processes in stable transplant conditions.

The dd-cfDNA fractions in the transplant recipients included here were consistent with a clinically stable post-transplant status, with fractions below 0.5%^29^ for KT and below 10% for LT,^17^ respectively. These findings not only validate the accuracy of our measurements but also suggest that the increased recipient cfDNA from various cell types in our cohort is not driven by allograft injury. As shown by Alnababteh et al.,^30^ early post-transplant increases in recipient-derived cfDNA correlated with %dd-cfDNA and predicted later adverse outcomes in lung transplant recipients, supporting a combined consideration of donor- and recipient-derived cfDNA when interpreting cfDNA profiles. Interestingly, some of the tissue-specific cfDNA release patterns identified in our study are consistent with the increased risk for complications associated with immunosuppressive treatments. CNIs are known to be nephrotoxic, causing chronic injury to kidney tubular cells and endothelial toxicity.^19^ The significant correlation between endothelial-derived cfDNA and cyclosporin A levels in our study may reflect CNI-induced vascular injury in these patients despite low dd-cfDNA levels. Elevated erythroid progenitor-derived cfDNA observed in KT recipients suggests differences in hematopoietic turnover between KT recipients and healthy individuals, in line with the common complication of post-transplant erythrocytosis in KT recipients due to persistently increased erythropoiesis.^31^ We also observed elevated pancreatic cfDNA in LT recipients, potentially related to previously described CNI-induced endocrine toxicity^32^^,^^33^ that may lead to new-onset diabetes mellitus after transplantation. Lastly, we demonstrated that hepatocyte cfDNA correlated with well-established biomarkers for liver injury in both the stable and early post-transplantation phases, supporting tissue-specific cfDNA as an injury marker. Differences in the strength of correlations between the different liver enzymes, particularly in the highly dynamic post-transplantation phase, may be related to the varying half-lives of these biomarkers.

For most stable LT recipients, the maintenance immunosuppression therapy consisted of either a CNI combined with mycophenolic acid/mycophenolate mofetil or a CNI combined with corticosteroids. In contrast, nearly all stable KT recipients received a triple-combination regimen comprising a CNI, mycophenolic acid/mycophenolate mofetil, and corticosteroids, reflecting a more aggressive immunosuppression. This difference in treatment may explain why stable LT recipients exhibited increased cfDNA release from certain cell types compared to stable KT recipients. More specifically, the elevated cfDNA levels observed across various tissues could indicate a heightened subclinical inflammatory state inadequately reflected in CRP levels. It is also conceivable that subclinical T cell-mediated rejection may contribute to these increases, as recently demonstrated.^34^ Further investigation into a potential systemic inflammatory state in different organ transplant recipients and its relationship with immunosuppressive regimens is thus warranted.

In the early post-transplantation phase, we demonstrated for the first time that a substantial fraction of dd-cfDNA in LT and KT recipients does not solely originate from the primary transplanted tissue. Particularly in KT recipients, dd-cfDNA levels in the first 24 h after transplantation were elevated with a subsequent decrease concordant with previous studies,^15^^,^^21^ whereas quantities of kidney epithelium-derived cfDNA were consistently low with no difference between the two time points. This observation that the primary transplanted tissue is not the major contributor to dd-cfDNA in KT recipients aligns with previous research in lung transplantation,^11^ showing that most dd-cfDNA early after transplantation was derived from immune cells. There are two potential explanations for this observation. On one hand, much more limited epithelial damage might occur through reperfusion than previously assumed based on dd-cfDNA measurements. Alternatively, cfDNA from kidney epithelium might predominantly be excreted through the urine, which could be investigated by the TOO analysis of urinary cfDNA in future studies. In this context, the lack of a difference in kidney epithelium cfDNA between KT recipients with and without DGF may also reflect a predominant release of kidney epithelial cfDNA directly into the urine.

In contrast to KT and lung transplantation,^11^ the majority of dd-cfDNA in LT recipients early after transplantation was derived from hepatocytes, and 50 to 130 h after transplantation, the percentage of dd-cfDNA closely matched the proportion of hepatocyte cfDNA. This finding contrasts with lung transplantation, where a significant contribution from non-lung tissues was reported to persist between 72 h and 7 days.^11^ Nevertheless, hepatocyte-derived cfDNA also did not account for all dd-cfDNA in LT in the first 24 h after transplantation. Although we cannot directly derive the TOO specifically for donor cfDNA fragments with our approach, the observed correlation between non-hepatocyte dd-cfDNA and granulocyte cfDNA may suggest a contribution of donor-derived granulocytes to dd-cfDNA early after liver transplantation. Similarly, the correlation of non-kidney epithelium dd-cfDNA in KT may indicate the release of cfDNA from kidney allograft endothelium. However, whether these cell and tissue types indeed contribute to dd-cfDNA early after transplantations would need to be confirmed with methods enabling donor- and recipient-specific TOO quantification.

Additional consideration should be given to the possibility that our findings reflect a combination of altered cfDNA release from specific cell types and impaired cfDNA clearance. Clearance of circulating cfDNA primarily occurs through phagocytosis by Kupffer cells in the liver,^35^^,^^36^^,^^37^ and CNIs have been shown to impair Kupffer cell function,^38^ which could lead to reduced clearance of cfDNA in transplant recipients under immunosuppression. Similarly, the high number of cell types in LT recipients showing a significant decrease between the first 24 h and 50 to 130 h after transplantation, which was not observed in KT recipients, may partly be the result of decreased hepatic cfDNA clearance early after LT. Furthermore, liver transplantation surgery, which typically involves more extensive skeletal muscle dissection and larger incisions than kidney transplantation, likely resulted in elevated levels of skeletal muscle cfDNA observed in LT but not KT recipients.

In conclusion, this study shows that our targeted deep sequencing assay can expand cfDNA analysis by integrating TOO information, offering insights into transplant recipient health beyond dd-cfDNA. The assay showed robust analytical performance and identified distinct cfDNA release patterns associated with different transplanted organ types. This lays a promising foundation for future investigations into the potential of cfDNA analysis as an early indicator of complications in the absence of clinical presentation. Future research expanding this approach to the investigation of complications, including rejection and drug toxicity, could pave the way for more personalized risk stratification and management of organ transplant recipients. Additionally, TOO analysis could be of interest in the context of novel therapies targeting CD38 to treat antibody-mediated rejection, in order to assess their impact on non-allograft tissues and cells. Finally, while this assay was applied in solid organ transplantation, TOO analysis is applicable in other clinical contexts to investigate the dynamics of tissue-specific cfDNA release and its potential as a diagnostic or prognostic biomarker.

### Limitations of the study

Although current clinical thresholds indicating kidney allograft rejection remain well above 0.3%, a limitation of our assay is its lower detection limit of 0.3% for TOO analysis, which resulted in no detection of kidney epithelium cfDNA in HC and stableKT recipients. Another limitation of the present study is the lack of samples collected during episodes of transplant complications, such as rejection, which would be valuable in future studies to both evaluate the clinical relevance of tissue-specific cfDNA dynamics and compare the integrated dd-cfDNA quantification to established diagnostic assays. Similarly, the robustness of the tissue specificity of the methylation markers across different disease states was not assessed here and could be investigated in future studies. Additionally, overlapping methylation signatures between cell types can lead to misclassification, as demonstrated by the detection of ovarian epithelium in some male participants, highlighting the potential benefit of further refinement of the cell type-specific markers in this assay. It is also important to note that while our assay validation relied on both in silico experiments and wet lab analyses, we did not assess TOO deconvolution specificity for all cell types. In addition, because some cell types, such as granulocytes, encompass multiple subpopulations with distinct functional roles, refining these markers to capture this heterogeneity may further enhance the diagnostic utility of cfDNA TOO analysis in transplant recipients and other clinical contexts. Future assay iterations could integrate additional markers beyond the initial 40 cell types, leveraging the expanding reference atlas data, to more accurately reflect the diversity of cell types contributing to cfDNA in patients with various pathologies.

## Resource availability

### Lead contact

Requests for further information and resources should be directed to and will be fulfilled by the lead contact, Ursula Amstutz (ursula.amstutz@insel.ch).

### Materials availability

This study did not generate new unique reagents.

### Data and code availability

• Raw adapter-trimmed FASTQ files generated in this study have been deposited at the European Genome-phenome Archive (EGA), which is hosted by the EBI and the CRG, under accession number EGAS50000000987.
• For the preprocessing, an analysis pipeline was implemented in Snakemake v7.22.0^39^ and is available at https://github.com/NichKu/TransMethylCFDx. The scripts used for dd-cfDNA calculation and in silico mixing were also made available there.
• Any additional information required to reanalyze the data reported in this paper is available from the lead contact upon request.

## Acknowledgments

The authors would like to thank all the patients and their families, as well as the healthy control volunteers, for their participation. We further thank all the University Institute of Clinical Chemistry and Clinical Genomics Lab technicians and staff of the Inselspital for their laboratory support, namely, Daniel Schärer, Gisela Andrey and Nicole Klaus. We also thank all nurses and medical staff involved in the sample collection and appreciate the support of Netanel Loyfer, who was the lead author of the publication on the methylation atlas. The study overview figure (Figure 1) in this manuscript was created with BioRender.com. This study was funded by the 10.13039/501100001711Swiss National Science Foundation, grant number 310030_188762, the Hemmi Foundation, and the Fondation Johanna Dürmüller-Bol. The biobanking was financially supported by the Department for Teaching and Research of the 10.13039/100018234Inselspital, 10.13039/100018234University Hospital of Bern, Bern, Switzerland.

## Author contributions

N.K. and U.A. designed the study. N.K. and F.S. recruited healthy control participants. D.S., V.B., and A.B. recruited patients with the help of F.S., while F.S. collected the clinical data. N.K. performed the research, acquired the data, and performed the data analysis. N.K. wrote the manuscript with U.A. C.K.Y.N., and C.R.L. provided scientific advice and helped with the interpretation of the data. All authors reviewed and approved the final version of the manuscript.

## Declaration of interests

The authors declare no competing interests.

## Declaration of generative AI and AI-assisted technologies in the writing process

During the preparation of this work, the author(s) used GPT-5.1 in order to improve the grammar and readability of the text. After using this tool/service, the author(s) reviewed and edited the content as needed and take(s) full responsibility for the content of the publication.

## STAR★Methods

### Key resources table

**Table:** 

REAGENT or RESOURCE	SOURCE	IDENTIFIER
**Biological samples**		

Whole blood, plasma, buffy coat	Inselspital, Bern University Hospital, Bern, Switzerland	https://www.insel.ch/de/
Purified primary hepatocytes	Biopredic International, Saint-Grégoire, France	Cat#HEP187332-TA05
Human Renal Cortical Epithelial Cells	PromoCell, Heidelberg, Germany	Cat#C-12660

**Critical commercial assays**		

K3EDTA blood collection tubes	Sarstedt, Nümbrecht, Germany	Cat#01.1605.001
QIAamp Circulating Nucleic Acid Kit	Qiagen, Hilden, Germany	Cat#55114
Qubit dsDNA 1X HS Assay Kit	Thermo Fisher Scientific, Waltham, MA, USA	Cat#Q33231
Bioanalyzer High Sensitivity DNA Kit	Agilent Technologies, Inc., Santa Clara, CA, USA	Cat#5067-4626
Dynabeads™ CD19 Pan B	Thermo Fisher Scientific, Waltham, MA, USA	Cat#11143D
Dynabeads™ HLA Class I	Thermo Fisher Scientific, Waltham, MA, USA	Cat#21002D
QIAamp DNA Blood Mini Kit	Qiagen, Hilden, Germany	Cat#51104
Clarefy DNA purification beads	Claret Bioscience, Santa Cruz, CA, USA	Cat#BD-001-96
NGS Methylation Detection System	Twist Bioscience, South San Francisco, CA, USA	Cat#103496
NEBNext Enzymatic Methyl-seq Conversion Module	New England Biolabs, Ipswich, MA, USA	Cat#E7125L
SRSLY NanoPlus kit	Claret Bioscience, Santa Cruz, CA, USA	Cat#K155B-96
PhiX Control v3	Illumina, San Diego, CA, USA	Cat#FC-110-3001

**Deposited data**		

Raw adapter-trimmed FASTQ files	This paper	EGA: EGAS50000000987

**Software and algorithms**		

TransMethylCFDx Analysis Pipeline	This paper	https://github.com/NichKu/TransMethylCFDx
Snakemake v7.22.0	Mölder et al.^39^	https://hub.docker.com/r/snakemake/snakemake
SRSLYumi v0.3	Claret Bioscience^40^	https://github.com/claretbio/SRSLYumi
bcl2fastq v2.20.0	Illumina, San Diego, CA, USA	https://emea.support.illumina.com/downloads/bcl2fastq-conversion-software-v2-20.html
Trim Galore v0.6.10	Krueger^41^	https://github.com/FelixKrueger/TrimGalore
bwa-meth v0.2.6	Pedersen et al.^42^	https://github.com/brentp/bwa-meth
UMI-tools v1.1.4	Smith et al.^43^	https://github.com/CGAOxford/UMI-tools
fgbio v2.1.0	Fulcrum Genomics fgbio^44^	https://github.com/fulcrumgenomics/fgbio
wgbstools v0.2.1	Loyfer et al.^45^	https://github.com/nloyfer/wgbs_tools
CollectHsMetrics v3.0.0	Broad Institute^46^	http://broadinstitute.github.io/picard/
SAMtools mpileup	Danecek et al.^47^	https://github.com/samtools/samtools

### Experimental model and study participant details

#### Study participants

The study was approved by the ethics committee of the Canton of Bern, Switzerland (2020-00953, 2019–00730), with procedures performed in accordance with the Declaration of Helsinki. Samples were obtained from participants who had provided written informed consent. A total of 40 liver and 53 kidney transplant recipients and 23 healthy control individuals were recruited at the Inselspital, Bern University Hospital, Switzerland, between 2020 and 2023. Information on the age and sex of the participants was recorded and the cohort demographics can be found in Table 1. No information on gender, ancestry, race, and ethnicity was collected. Exclusion criteria for transplant recipients were: being under 18 years of age, no blood sampling feasible at any of the intended time points and recipients of both kidney and liver or any other organ transplant. Exclusion criteria for healthy control participants were: being under 18 years of age, current or recent (within 3 months) pregnancy, a history of organ transplantation, current or recent (within 3 months) blood transfusion or administration of plasma-derived products, or a current known malignancy. Two cohorts of transplant recipients were included with kidney or liver allografts, one consisting of stable recipients, and the second cohort included patients with samples collected in the first week after transplantation (early post-transplantation cohort).

LT and KT recipients were considered stable based on a combination of clinical and laboratory criteria. All laboratory parameters used for classification were required to meet the stability criteria for measurements obtained between at least 30 days post-transplantation and at least one month after the corresponding sample collection. LT recipients were classified as stable if they had not received any organ other than a liver, had ASAT and ALAT values below 35 U/L (upper reference limit), showed a coefficient of variation below one for blood leukocyte counts and did not undergo a for-cause biopsy within three months after sample collection. KT recipients were classified as stable if they had not received any organ other than a kidney, did not require dialysis after 30 days post-transplantation, had a chronic kidney disease stage below G3b, maintained an estimated glomerular filtration rate (eGFR) above 30 mL/min/1.73 m^2^ (allowing for one measurement below 30), showed a coefficient of variation below one for blood leukocyte counts and did not undergo a for-cause biopsy within three months after sample collection. LT and KT recipients who underwent re-transplantation within one year post-transplantation were excluded from the stable group.

Clinical data and laboratory parameters were extracted from the patients’ electronic health records. The time in hours after transplantation for the early-post transplantation cohort was calculated as the time since the final surgical suture timestamp, extracted from the patient’s electronic health record.

#### Primary human cells

Cryopreserved purified hepatocytes in suspension from a single 73-year-old Caucasian female donor were ordered from Biopredict International (Saint-Grégoire, France). Purified human renal cortical epithelial cells (HRCEpC) from a single 50-year-old Caucasian male donor suspended in RNAlater (Thermo Fisher Scientific, Waltham, MA, USA) were obtained from PromoCell (Heidelberg, Germany), hereafter referred to as primary kidney cells. According to the supplier, the primary kidney cells originated from the renal cortex, represent epithelial populations from tubular and corpuscular structures as well as collecting ducts, and stained positive for cytokeratin.

### Method details

#### Sample collection and cfDNA isolation

Venous blood samples were collected between the first day and up to 12 months after the transplantation from the transplant recipients. From the healthy control individuals, venous blood samples were collected at a single appointment. Up to 30 mL of whole blood from healthy controls and up to 15 mL from transplant recipients were collected in K3EDTA blood collection tubes (Sarstedt, Nümbrecht, Germany) and processed within 2 hours after collection. The blood samples were centrifuged at 2’000 g for 15 min. at room temperature (RT). The plasma was separated and re-centrifuged at 3’800 g for 10 min. at RT. The plasma supernatant and up to 1 mL of buffy coat were frozen following a pre-programmed freezing curve and subsequently stored at −80 °C in the Liquid Biobank Bern (www.biobankbern.ch) until DNA extraction.

For cfDNA isolation, the plasma was thawed at RT and isolated from 5 mL using the QIAamp Circulating Nucleic Acid Kit (Qiagen, Hilden, Germany). DNA concentration was measured using the Qubit dsDNA 1X HS Assay Kit (Thermo Fisher Scientific, Waltham, MA, USA). Samples showing markedly higher cfDNA concentrations compared with others in the corresponding group were additionally analyzed using the 2100 Bioanalyzer and Bioanalyzer High Sensitivity DNA Kit (Agilent Technologies, Inc., Santa Clara, CA, USA) to confirm the expected cfDNA size distribution and exclude potential genomic DNA contamination.

#### Primary human cell and genomic DNA isolation

Purified primary human cells from the liver, kidney, and immune system were used to validate the targeted methylation panel. B cells were isolated from whole blood of two healthy control individuals using Dynabeads™ CD19 Pan B (Thermo Fisher Scientific, Waltham, MA, USA). T cells were isolated from whole blood of a healthy control individual using Dynabeads™ HLA Class I (Thermo Fisher Scientific, Waltham, MA, USA). The HLA Class I beads specifically bind to the CD8 membrane antigen on human cells and hence isolate CD8^+^ T lymphocytes. The differential CBC of whole blood from healthy control individuals was determined using the XN-1000 Hematology Analyzer (Sysmex Suisse AG, Horgen, Switzerland) before genomic DNA (gDNA) extraction. The buffy coat was removed from the ETDA blood sample from healthy control individuals after the first centrifugation at 2’000 g. The buffy coat was washed twice by resuspension in 10 mL PBS (no Mg^2+^ or Ca^2+^) and subsequently pelleted at 300 g for 15 min., followed by a final resuspension in 1.5 mL of PBS. The gDNA was extracted from 200 μl primary human kidney cell suspension, primary human hepatocyte cell suspension, whole blood, buffy coat cell suspensions and suspensions of cells bound to the Dynabeads™ using the QIAamp DNA Blood Mini Kit (Qiagen, Hilden, Germany). DNA concentration was measured using the Qubit dsDNA 1X HS Assay Kit (Thermo Fisher Scientific, Waltham, MA, USA).

The gDNA was sheared in 20 μl 10 mM Tris-Cl (pH 8.5) to 170 bp using the COVARIS LE220+ ultrasonicator (Covaris Inc., Woburn, MA, USA). The sheared gDNA from the pure primary cell types was size-selected to resemble the average profile of cfDNA. For that, Clarefy DNA purification beads (Claret Bioscience, Santa Cruz, CA, USA) were added at 0.7x ratio, incubated for 10 min. and placed on a magnetic rack. The supernatant was transferred to a new tube, beads were added at a 1.2x ratio, incubated for 10 min. and placed on a magnetic rack. The supernatant was discarded, the beads were washed twice with 80% ethanol and the DNA was eluted in 32 μl nuclease-free water. DNA concentration was measured using the Qubit dsDNA 1X HS Assay Kit (Thermo Fisher Scientific, Waltham, MA, USA).

#### Panel design

A hybridization capture panel was designed to target cell type-specific hypomethylated regions for TOO analysis and SNPs for dd-cfDNA quantification. The panel design for TOO analysis was based on cell-type-specific markers for 40 different cell types published by Loyfer et al.,^12^ including data from 39 cell types and megakaryocyte-specific data.^48^ The markers were selected as blocks of CpGs with similar methylation levels across three or more CpGs in the human genome that are differentially methylated between the target and the background cell types.

The panel was developed using a stepwise design approach: For the methylation marker selection, cell type-specific markers were ranked as previously described^12^ based on the difference in the average methylation between the target cell type and other cell types. Markers were filtered to exclude regions with complete overlap with Alu repeat annotations^49^^,^^50^ to minimize off-target binding of the capture probes. SNPs were selected for dd-cfDNA fraction determination based on the following criteria: 1. minor allele frequency (MAF) between 0.45 and 0.55,^51^ 2. absence of G and C alleles in the databases,^51^ 3. non-overlapping with short interspersed nuclear elements (SINEs) annotations,^52^ and 4. distributed across all 22 autosomes.

Capture probes were designed by Twist Bioscience (South San Francisco, CA, USA) against hypermethylated (100% methylated CpGs) and hypomethylated (100% CpGs) fragments from the target regions, focusing on achieving high coverage of the marker CpG sites. We further refined the design by evaluating the coverage of the markers by probes using varying probe tiling densities (1x, 1.5x and 2x). This process aimed to optimize the trade-off between maximizing CpG site coverage and minimizing redundant capture of non-essential regions. A tiling density of 1.5x was chosen. To evaluate the design, the regions were manually inspected and markers where CpG sites were inadequately covered by probes, assuming a mean library insert size of 167 bp, were omitted from the panel. All SNPs were fully covered by probes.

The NGS Methylation Detection System (Twist Bioscience, South San Francisco, CA, USA) platform was used and 120 bp biotinylated probes were synthesized by Twist Bioscience. Probes with increased off-target rates were excluded from the final panel design.

The final panel design targeted 975 cell type-specific markers (Table S1) with a median marker length of 283 bp (IQR: 163–445 bp) and a median of 7 CpGs (IQR: 6–10 CpGs) per marker. Regarding the SNPs, the first version of the panel targeted 117 SNPs, with 115 on all 22 autosomes and two on the X chromosome. A second version of the panel was designed that included 302 SNPs (Table S2), with 300 on all 22 autosomes and two with one on each sex chromosome. While some samples were analyzed with the first version of the panel and the rest with the second version, equivalent performance was shown (Figure S16). The SNPs on the sex chromosomes were not used for %dd-cfDNA quantification but for quality control purposes only. The final panel design covered a total target region of approximately 497 kbp.

#### Targeted methylation sequencing

For sequencing, up to 150 ng of sheared gDNA or up to 70 ng of cfDNA was converted using the NEBNext Enzymatic Methyl-seq Conversion Module (New England Biolabs, Ipswich, MA, USA) by oxidizing 5-mC to 5-hmC and subsequently deaminating unmethylated cytosines. The clean-up steps were performed using Clarefy DNA purification beads (Claret Bioscience, Santa Cruz, CA, USA). The enzymatic conversion protocol was adapted to maximize recovery by adding 100 μl of purification beads for the first and 170 μl for the second clean-up. The DNA was eluted in 16 μl nuclease-free water after the first clean-up. The converted DNA after the second clean-up was eluted in 18 μl and the whole eluate was immediately used as input for library preparation with the SRSLY NanoPlus kit (Claret Bioscience, Santa Cruz, CA, USA). Unique molecular identifiers (UMI) and unique dual indices (UDI) were used, and 8–10 index PCR cycles were performed depending on the original input, as instructed in the manual. The DNA library concentration was quantified using the Qubit dsDNA 1X HS Assay Kit (Thermo Fisher Scientific, Waltham, MA, USA), while the quality and fragment size distribution were analyzed with the Agilent High Sensitivity DNA Kit (Agilent Technologies Inc., Santa Clara, CA, USA). The libraries were stored at −20°C until target enrichment. Up to 14 libraries were multiplexed and target enrichment was performed with up to 2 μg total input per reaction using the Twist NGS Methylation Detection System (Twist Bioscience, San Francisco, CA, USA). The probes were hybridized to the targets for two hours and 15 post-capture PCR cycles were performed. Final concentration, quality, and fragment size were determined as described above. Up to five enriched library pools were combined, spiked with PhiX v3 library (Illumina, San Diego, CA, USA) at 10% and sequenced with 150 bp paired-end reads on a NextSeq 550 or NovaSeq 6000 (Illumina, San Diego, CA, USA).

#### ddPCR dd-cfDNA and total cfDNA quantification

To verify the dd-cfDNA quantification using the integrated targeted sequencing method, the fractional abundance and absolute GE/mL plasma of dd-cfDNA were determined in a subset of samples as previously described^18^ using ddPCR and assays targeting mismatches between the donor and recipient in the *HLA-DRB1* gene. For ddPCR-based dd-cfDNA quantification, the total amount of cfDNA was determined by measuring the copy number of the gene *RPP30* using a previously published assay^53^ following the same ddPCR protocol as for the *HLA-DRB1* assays.

#### Bioinformatic analysis and sequencing panel-based dd-cfDNA quantification

Demultiplexing and UMI sequence extraction were performed using SRSLYumi v0.3^40^ and the bcl2fastq v2.20.0 software. Adapter and quality trimming was performed with Trim Galore v0.6.10^41^ and the reads were mapped to the human genome hg19 using bwa-meth v0.2.6.^42^ The mapped reads were grouped based on their genomic coordinate and UMI with UMI-tools v1.1.4^43^ and consensus reads were created from groups with one or more reads using fgbio v2.1.0.^44^ The consensus reads were remapped, filtered (mapping quality ≥50), stripped from non-CpG nucleotides and converted to PAT and BETA files using wgbstools v0.2.1.^45^ The TOO deconvolution was performed using a fragment-level non-negative least squares-based algorithm as previously described^12^ using reads with ≥4 CpGs.

The cytosine conversion efficiency was determined based on the average methylation rate of non-CpG-context cytosines. The target region coverage was determined using the Picard tool’s CollectHsMetrics v3.0.0.^46^

To determine the fraction of dd-cfDNA, the number of filtered consensus reads containing each allele per SNP was calculated using SAMtools mpileup.^47^ The %dd-cfDNA for each SNP was computed as the count of the alternate allele divided by the total allele count. For samples with dd-cfDNA fractions exceeding 20%, the recipient’s genotype was employed to identify and exclude heterozygous SNPs in the recipient. Subsequently, the recipient’s genotype was utilized to identify the donor allele for each SNP where the recipient was homozygous and a second allele was detected. The recipient genotype was determined by sequencing gDNA from the recipient’s buffy coat. For fractions ≤20%, SNPs with %dd-cfDNA between 30% and 70% were assumed to be recipient heterozygous and excluded. Outliers, defined as SNPs with %dd-cfDNA >2 standard deviations from the mean %dd-cfDNA, were removed.

For samples with a mean %dd-cfDNA >10%, donor genotypes were incorporated to adjust estimates by identifying donor homozygous and heterozygous SNPs. To identify donor genotypes, a Gaussian mixture model assuming the three components was applied, implemented in scikit-learn v1.3.0, enabling the classification of SNPs as donor homozygous for the reference allele, homozygous for the alternate allele or heterozygous. The mean of SNPs where both donor and recipient were homozygous for the same allele was subtracted from the adjusted mean to account for background.

Absolute GE/mL for cfDNA TOO and dd-cfDNA were determined by multiplying the respective fractions obtained by targeted sequencing with total cfDNA concentration (ng/mL) measured using Qubit, multiplied by a factor of 303 (the haploid human genome weight in pg) to convert ng/mL to GE/mL. Absolute dd-cfDNA concentrations derived from Qubit-based total cfDNA quantification showed strong correlation with ddPCR-based total cfDNA measurements in a subset of samples, confirming the consistency of both absolute quantification approaches (Figure S17).

#### *In silico* mixtures

For TOO analysis based on in silico mixtures, hepatocyte gDNA reads were combined with a background of WBC gDNA reads from three healthy control individuals. In contrast, kidney gDNA reads were mixed into a mixture consisting of 97% WBC and 3% hepatocyte gDNA reads (for a plasma-like background). A shuffled subset of both hepatocyte and kidney gDNA reads was mixed into the background at various fractions ranging from 90% to 0%. All mixtures were generated with a target region coverage ranging from 30x to 1000x. For each mixture and coverage combination, 20 replicates were generated. Read mixing was performed using wgbstools v0.2.1.^45^ For the SNP-based dd-cfDNA quantification in silico mixtures, plasma cfDNA reads from one healthy control individual sample were mixed into a background of reads from a second healthy control individual sample at various fractions between 90% and 0%. The target region coverage ranged from 200x to 1000x. For each mixture and coverage combination, 20 replicates were generated. Read mixing was performed using SAMtools.^47^

### Quantification and statistical analysis

All statistical analyses and graphs were performed and created using Python v3.10. Global statistical testing per cell type between three groups was conducted using the non-parametric Kruskal-Wallis test with P values adjusted across cell types using the Benjamini-Hochberg procedure, with the false discovery rate (FDR) threshold set at 0.05 to control for the type I error. For significant global tests, Dunn’s post-hoc test was employed to identify pairwise differences. Dunn’s post-hoc P values were adjusted for multiple testing across groups using the Bonferroni method. To assess differences between two groups, the Wilcoxon signed-rank test was utilized for paired samples, while the Mann–Whitney U test was applied for unpaired samples. Multiple hypothesis testing, without preceding global testing, was corrected using the Benjamini-Hochberg procedure. *P* values were deemed significant when less than 0.05. For correlation analysis, Pearson’s correlation coefficient was calculated.

## Supplemental information

Document S1. Figures S1–S17

Table S1. Tissue-of-origin methylation marker hg19 coordinates

Table S2. Single-nucleotide polymorphism marker hg19 coordinates
