# Supplementary material for: Integrated targeted sequencing reveals unique tissue-of-origin and donor-derived cell-free DNA signatures in stable organ transplant recipients
Source: iScience. 2026 Mar 30;29(5):115315. doi: 10.1016/j.isci.2026.115315 (PMC13092851; doi:10.1016/j.isci.2026.115315)
Supplement: Document S1. Figures S1–S17 [file mmc1.pdf]

iScience, Volume *29*

Supplemental information

Integrated targeted sequencing reveals unique tissue-of-origin and donor-derived cell-free DNA signatures in stable organ transplant recipients

Nicholas Kueng, Fanny Sandberg, Daniel Sidler, Vanessa Banz, Annalisa Berzigotti, Charlotte K.Y. Ng, Carlo R. Largiadèr, and Ursula Amstutz

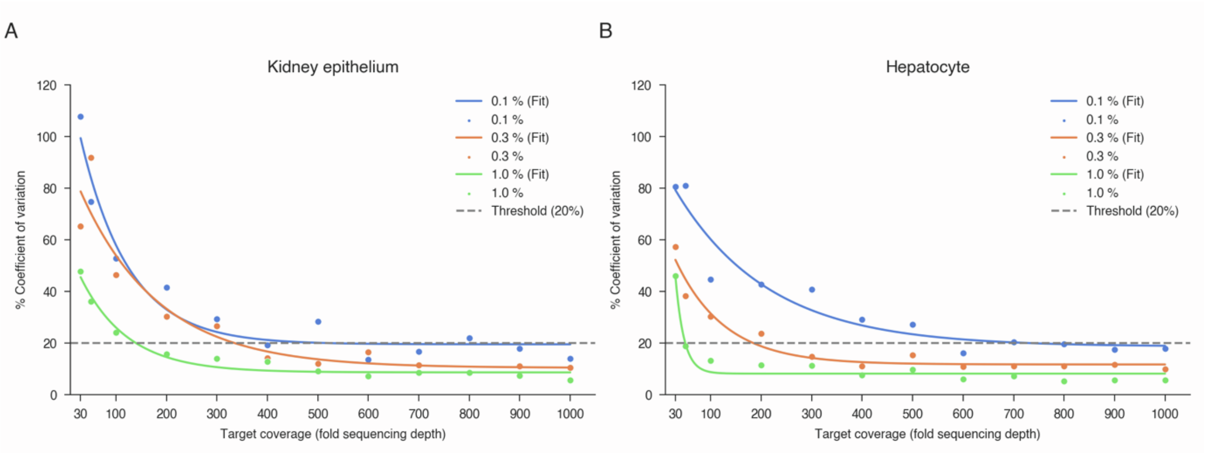

Figure S1. Coefficient of variation for in silico mixtures for different tissue proportions and target region depth of coverage, related to STAR Methods.
Reads from sequenced genomic DNA from kidney epithelium (**A**) or hepatocytes (**B**) samples were computationally mixed into a background of leukocyte genomic DNA reads from three individuals at 0.1, 0.3 and 1.0% fractions. The background for the kidney epithelium (**A**) mixtures consisted of 97% leukocyte and 3% hepatocyte reads. The mixtures were produced for different target-region depth-of-coverage (x-axis). The y-axis indicates the coefficient of variation (CV), calculated across 20 replicates. The dashed grey line represents the threshold for a CV of 20%.

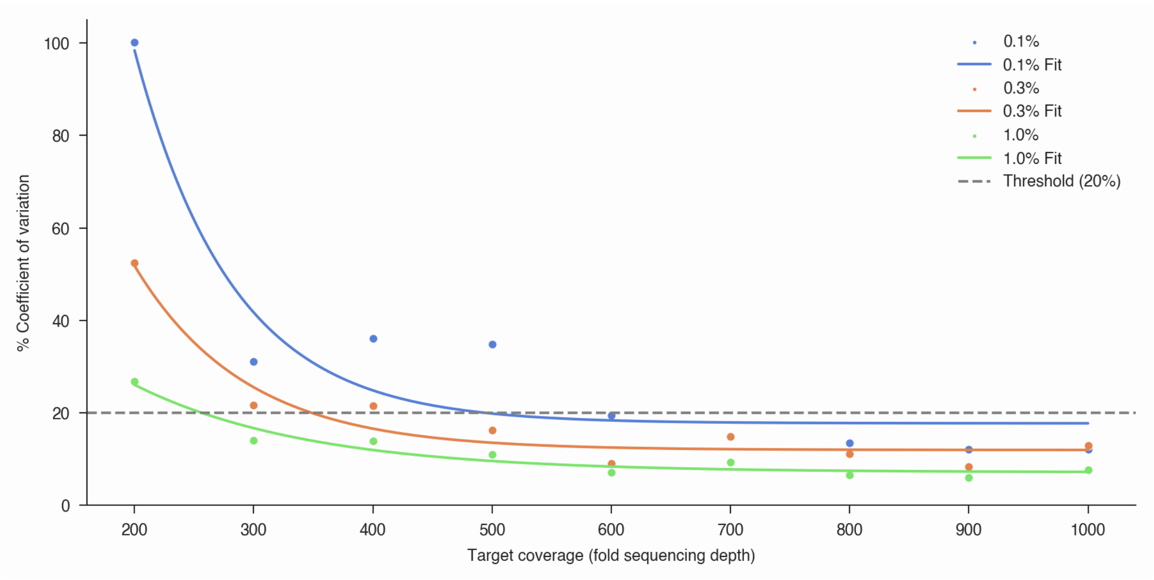

Figure S2. Coefficient of variation for in silico mixtures for different donor-derived cfDNA proportions and target region depth of coverage, related to STAR Methods.
Plasma cfDNA reads from two healthy controls were mixed at 0.1, 0.3 and 1.0% fractions. The mixtures were produced for different target-region depth-of-coverage (x-axis). The y-axis shows the coefficient of variation (CV), calculated across 20 replicates. The grey line represents the threshold for a CV of 20%.

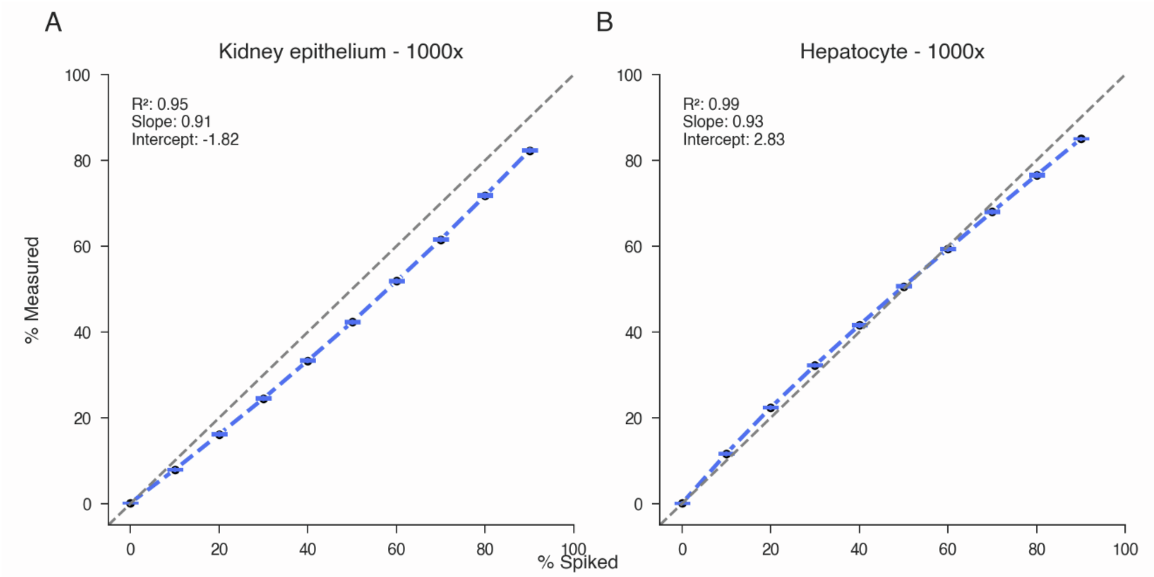

Figure S3. Linearity of tissue-of-origin deconvolution, related to STAR Methods.
Reads from sequenced genomic DNA from kidney epithelium (**A**) or hepatocytes (**B**) samples were computationally mixed into a background of leukocyte genomic DNA reads from three healthy control individuals. The background for the kidney epithelium (**A**) mixtures consisted of 97% leukocyte and 3% hepatocyte reads. Mixtures were performed at a sequencing depth of 1’000x. Black markers show the median determined contribution for 20 replicates, with the error bars displaying one standard deviation. The grey line represents the identity line (y=x).

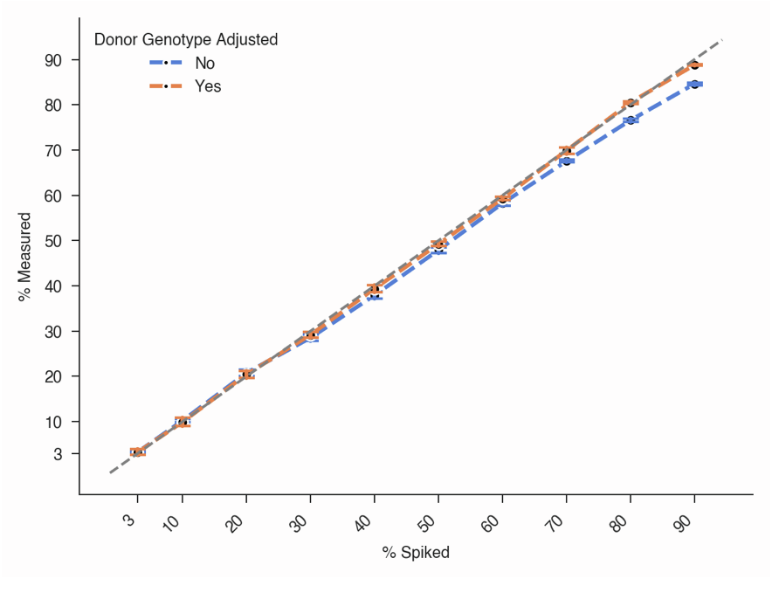

Figure S4. Linearity of dd-cfDNA quantification, related to STAR Methods.
Plasma cfDNA reads from two healthy controls were mixed at various fractions. Mixtures were performed at a sequencing depth of 1’000x. Black markers show the median determined contribution for 20 replicates, with the error bars displaying one standard deviation. The grey line represents the identity line (y=x).

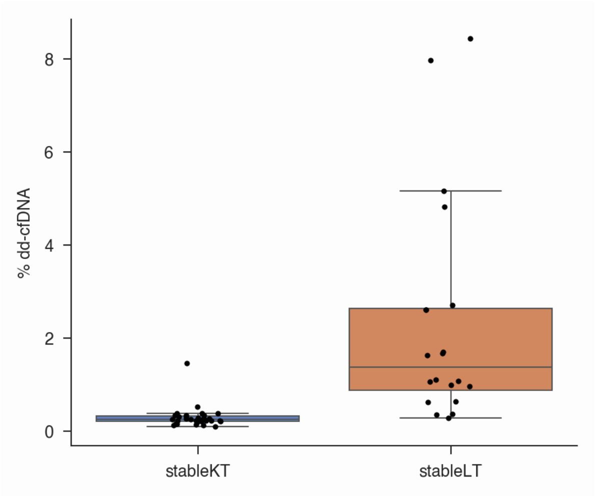

Figure S5. %dd-cfDNA in stable transplant recipients, related to Figure 3.
Boxplot illustrating the %dd-cfDNA levels in stable transplant recipients. StableLT: stable liver transplant recipients (n = 20), stableKT: stable kidney transplant recipients (n = 31). The box spans the 25th to 75th percentiles with a line at the median. Whiskers extend to 1.5× the interquartile range.

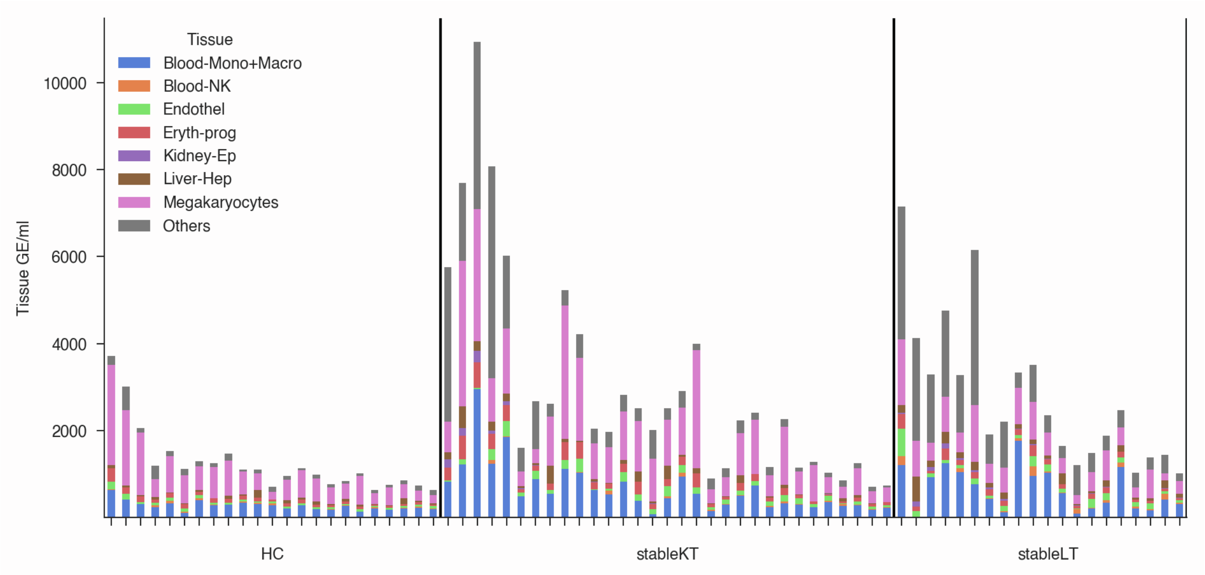

Figure S6. Absolute cfDNA per cell type for stable transplant recipients versus healthy controls, excluding granulocyte cfDNA, related to Figure 3.
Absolute tissue-of-origin GE/mL per individual sample, with bars grouped by cohort and in descending order of total GE/mL. Each colored segment represents a distinct cell type (without granulocytes) or cell type group. StableLT: stable liver transplant recipients (n = 20), stableKT: stable kidney transplant recipients (n = 31), HC: healthy control group (n = 23).

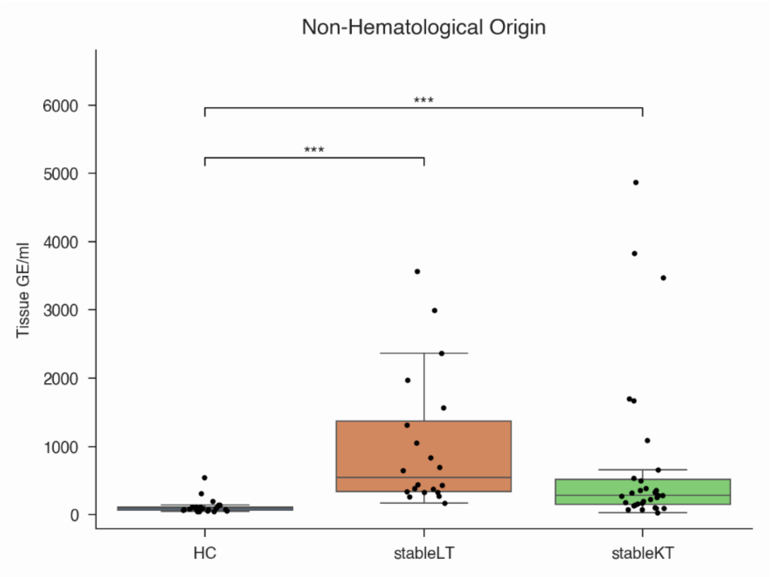

Figure S7. Absolute cfDNA of non-hematological origin in stable transplant recipients versus healthy controls, related to Figure 3.
Boxplot illustrating the absolute cfDNA levels from non-hematological sources across different groups. StableLT: stable liver transplant recipients (n = 20), stableKT: stable kidney transplant recipients (n = 31), HC: healthy control group (n = 23). The Kruskal-Wallis test was used for global testing and Dunn's post-hoc test for multiple pairwise comparisons between the groups. Dunn's test P-values were adjusted using the Bonferroni correction to control for multiple comparisons. *** P < 0001, no comparison annotation means P > 0.05. The box spans the 25th to 75th percentiles with a line at the median. Whiskers extend to 1.5× the interquartile range.

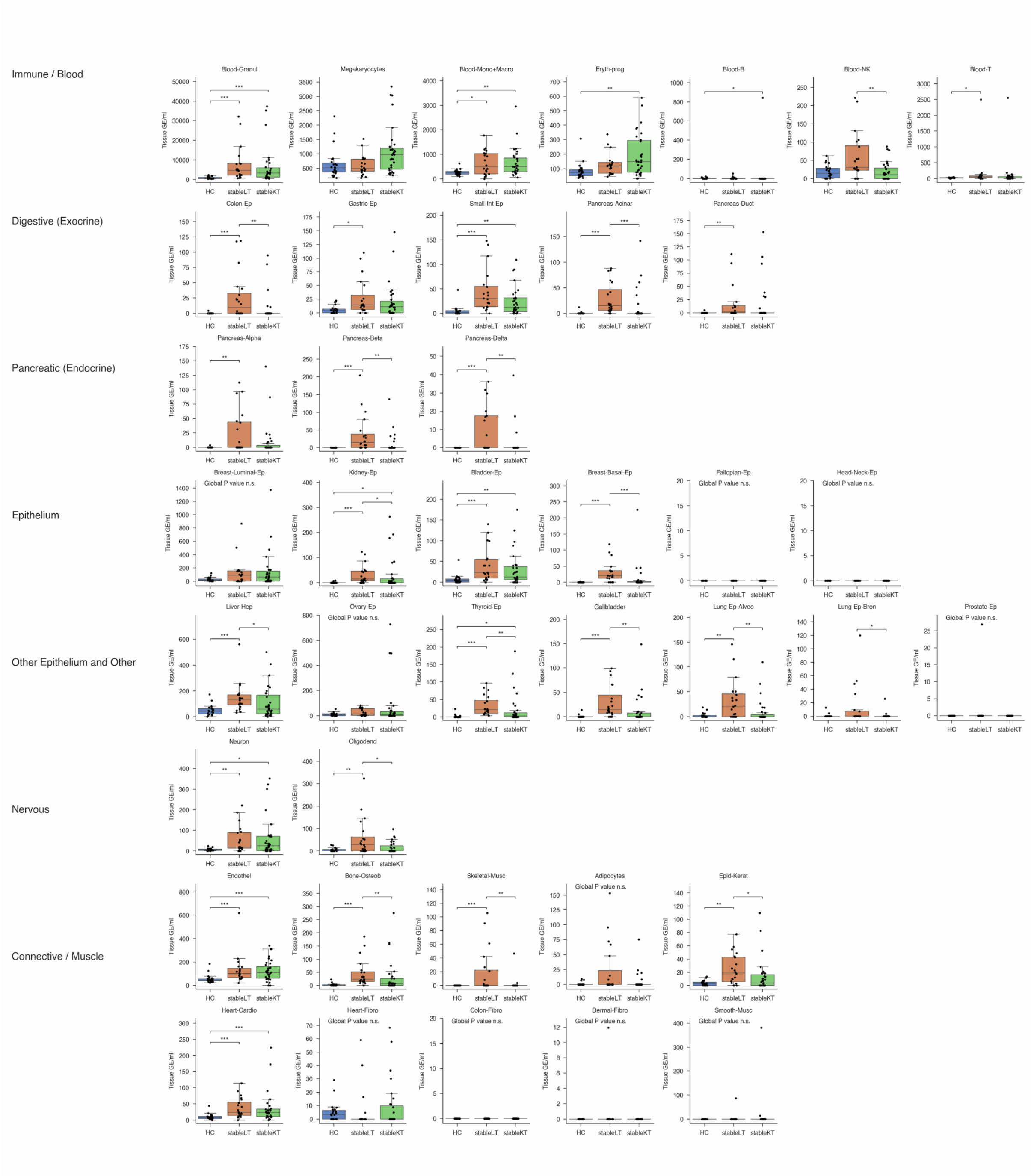

Figure S8. Absolute cfDNA tissue-of-origin from all 40 cell types for stable transplant recipients versus healthy controls, related to Figure 3.

Boxplots of absolute cfDNA from all cell types by cohort group. StableLT: stable liver transplant recipients (n = 20), stableKT: stable kidney transplant recipients (n = 31), HC: healthy control group (n = 23). For each cell type, the Kruskal-Wallis test was performed for global comparisons, with the Benjamini-Hochberg procedure applied to control the false discovery rate (FDR) across cell types. For significant results, Dunn's post-hoc test was used for pairwise comparisons between groups, and its P-values were adjusted using the Bonferroni correction to account for multiple comparisons between groups for a given cell type. * $P < 0.05$, ** $P < 0.01$, *** $P < 0.001$, **** $P < 0.0001$, no comparison annotation means $P > 0.05$. n.s. means not statistically significant. The box spans the 25th to 75th percentiles with a line at the median. Whiskers extend to 1.5× the interquartile range.

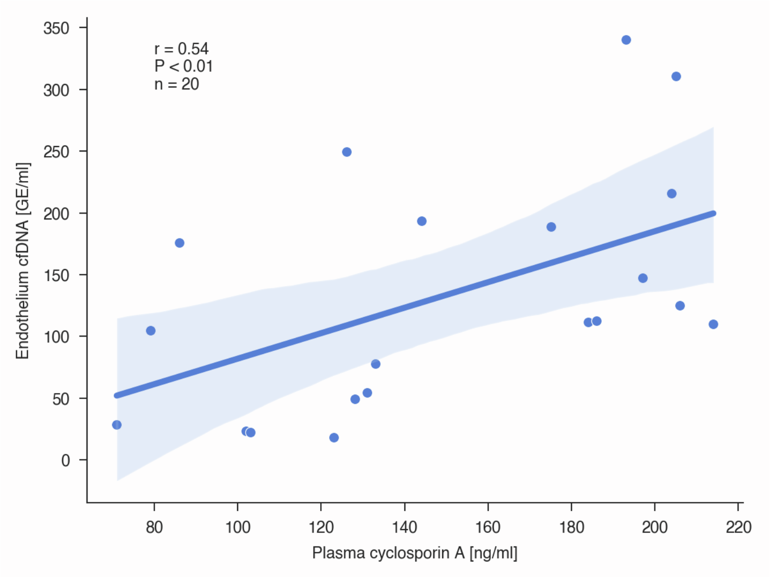

Figure S9. Correlation between absolute endothelium cfDNA versus matched plasma cyclosporin A levels, related to Figure 3.
The blue line represents the linear regression with its corresponding 95% confidence interval. Shown is Pearson’s r correlation coefficient.

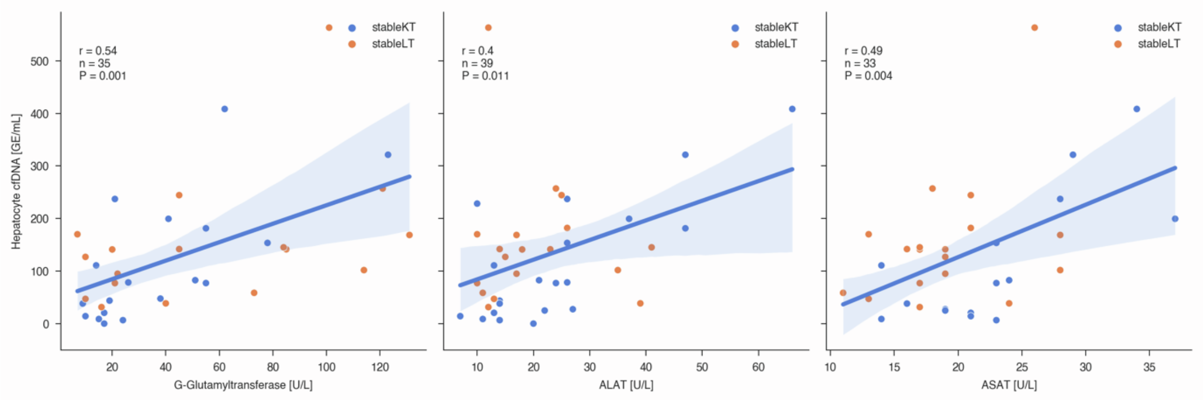

Figure S10. Correlation between absolute hepatocyte cfDNA versus liver enzyme measurements in the stable transplant cohorts, related to Figure 3.
The blue lines represent the linear regression with its corresponding 95% confidence interval. Shown is Pearson’s r correlation coefficient. stableLT: stable liver transplant recipients, stableKT: stable kidney transplant recipients.

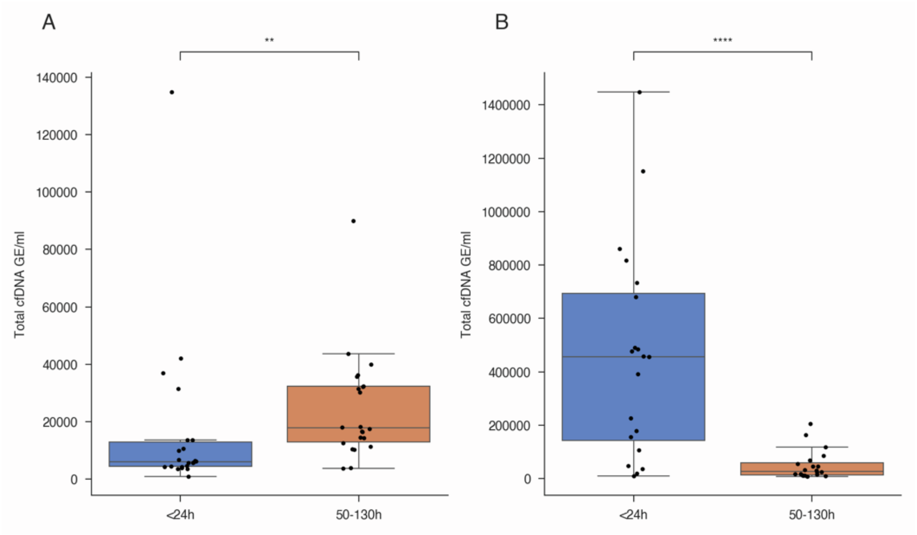

Figure S11. Total cfDNA early post-transplantation, related to Figure 4.
Boxplot of total cfDNA for (**A**) kidney transplant (n = 22) and (**B**) liver transplant recipients (n = 20) early post-transplantation. Time on the x-axis indicates the time since the transplantation, in which the samples were collected. The Wilcoxon signed-rank test was used. ** P < 0.01, *** P < 0.001. The box spans the 25th to 75th percentiles with a line at the median. Whiskers extend to 1.5× the interquartile range.

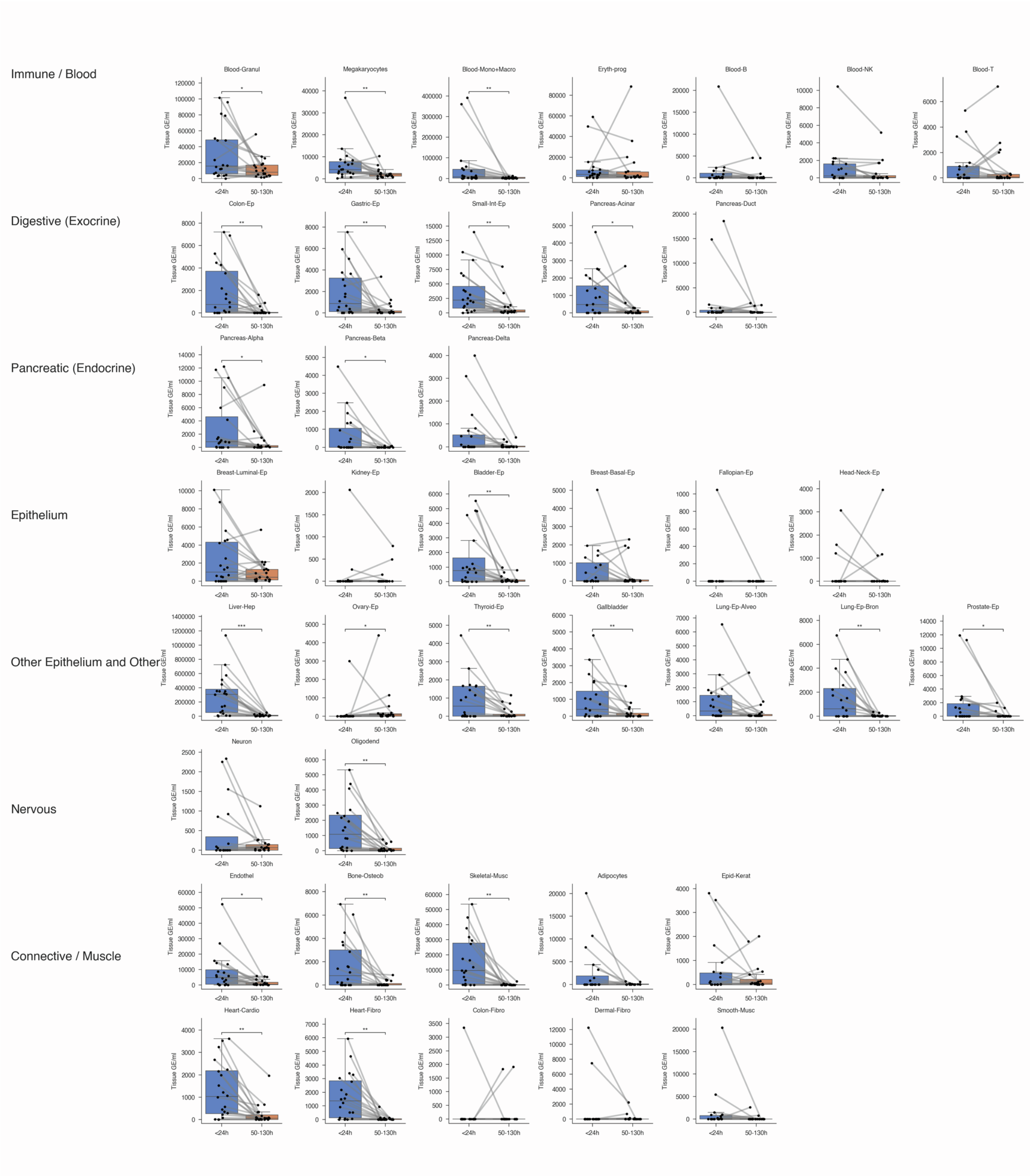

Figure S12. Absolute cfDNA tissue-of-origin from all 40 cell types for liver transplant recipients at two time points, early post-transplantation, related to Figure 4.
Boxplot of absolute cfDNA from all cell types by time point. The Wilcoxon signed-rank test was used, and P-values were adjusted for multiple comparisons across cell types using the Benjamini-Hochberg procedure to control the false discovery rate (FDR). * P < 0.05, ** P < 0.01, *** P < 0.001, **** P < 0.0001, no comparison annotation means P > 0.05. The box spans the 25th to 75th percentiles with a line at the median. Whiskers extend to 1.5× the interquartile range.

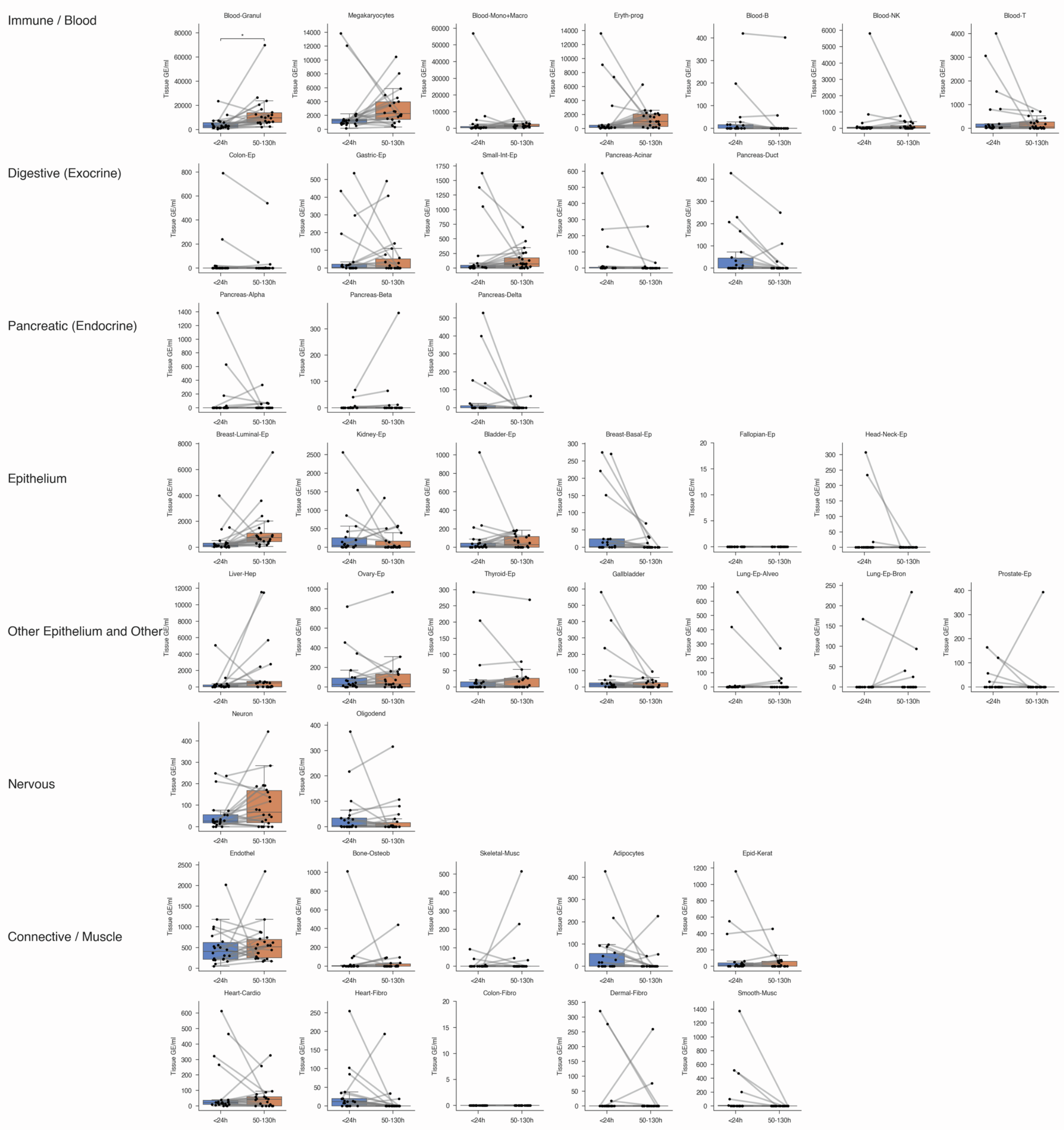

Figure S13. Absolute cfDNA tissue-of-origin from all 40 cell types for kidney transplant recipients at two time points, early post-transplantation, related to Figure 4.

Boxplot of absolute cfDNA from all cell types by time point. The Wilcoxon signed-rank test was used, and P-values were adjusted for multiple comparisons across cell types using the Benjamini-Hochberg procedure to control the false discovery rate (FDR). * $P < 0.05$, no comparison annotation means $P > 0.05$. The box spans the 25th to 75th percentiles with a line at the median. Whiskers extend to 1.5× the interquartile range.

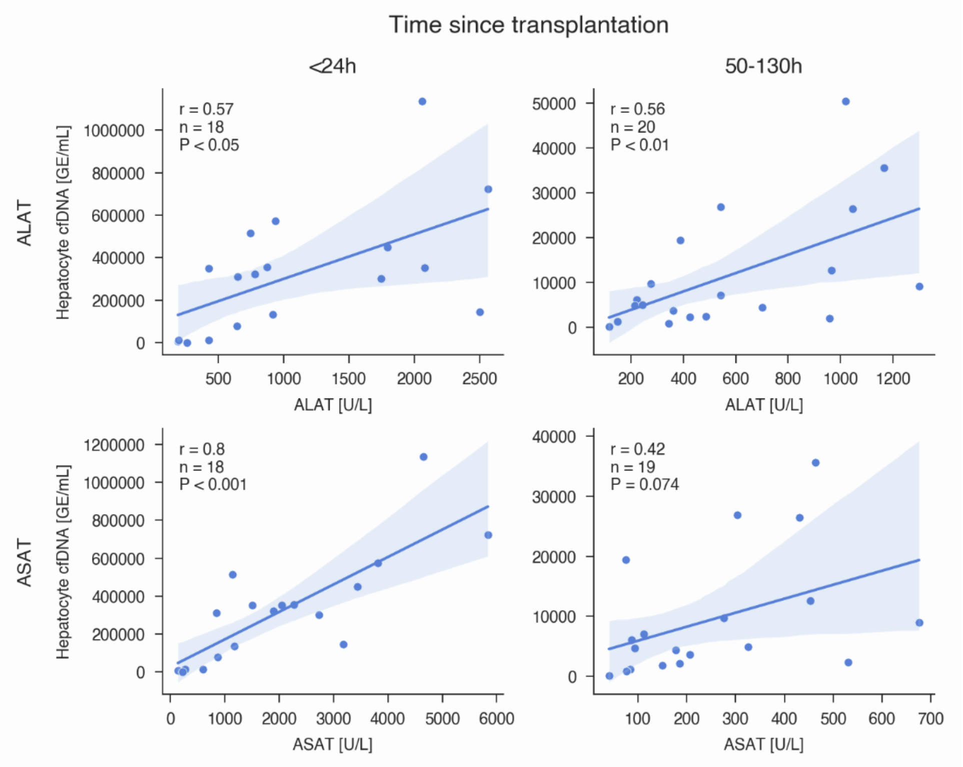

Figure S14. Correlation between absolute hepatocyte cfDNA and liver enzyme measurements in liver transplant recipients from the early post-transplantation cohort, related to Figure 4.
The blue lines represent the linear regression with its corresponding 95% confidence interval. Shown is Pearson's r correlation coefficient.

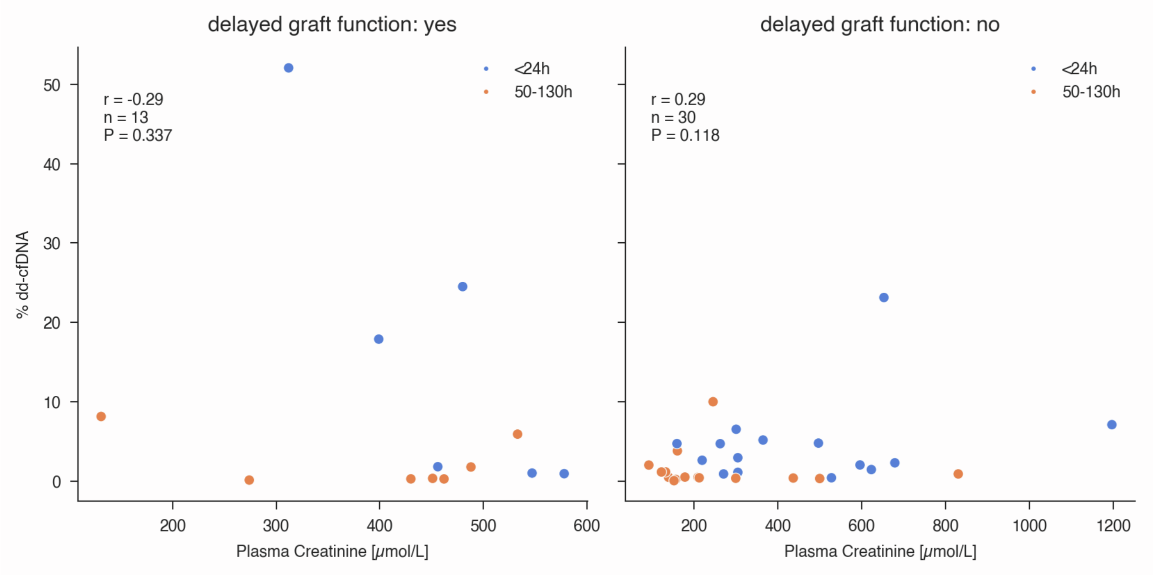

Figure S15. Correlation between plasma creatinine and %dd-cfDNA in kidney transplant recipients from the early post-transplantation cohort with and without delayed graft function, related to Figure 4.
Shown is Pearson's r correlation coefficient. The color of the dots indicates the time point post-transplantation.

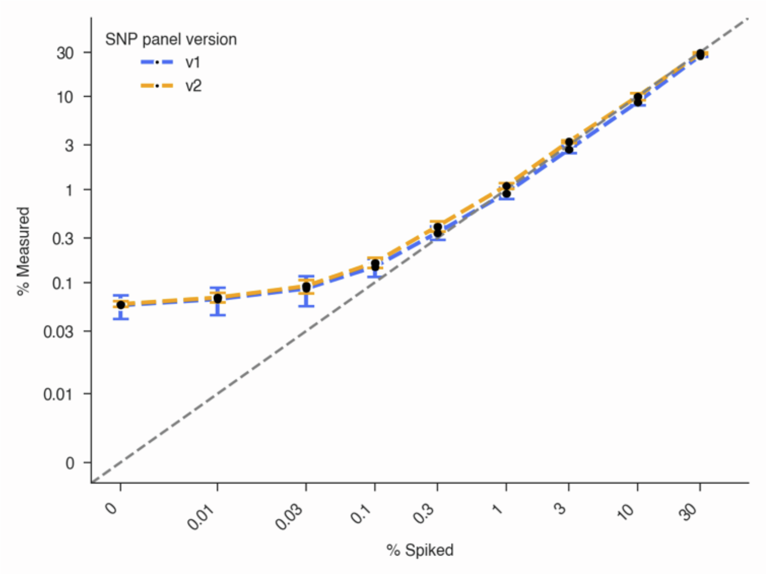

Figure S16. Comparability of dd-cfDNA quantification with two SNP panel versions, related to STAR Methods.
Plasma cfDNA reads from two healthy controls were mixed at various fractions. Mixtures were performed at a sequencing depth of 1'000x. dd-cfDNA fractions were calculated based on the SNPs included either in panel version v2 or with the selection of SNPs from v1, a subset of v2. Black markers show the median determined dd-cfDNA fraction for 20 replicates, with the error bars displaying one standard deviation. The grey line represents the identity line (y=x).

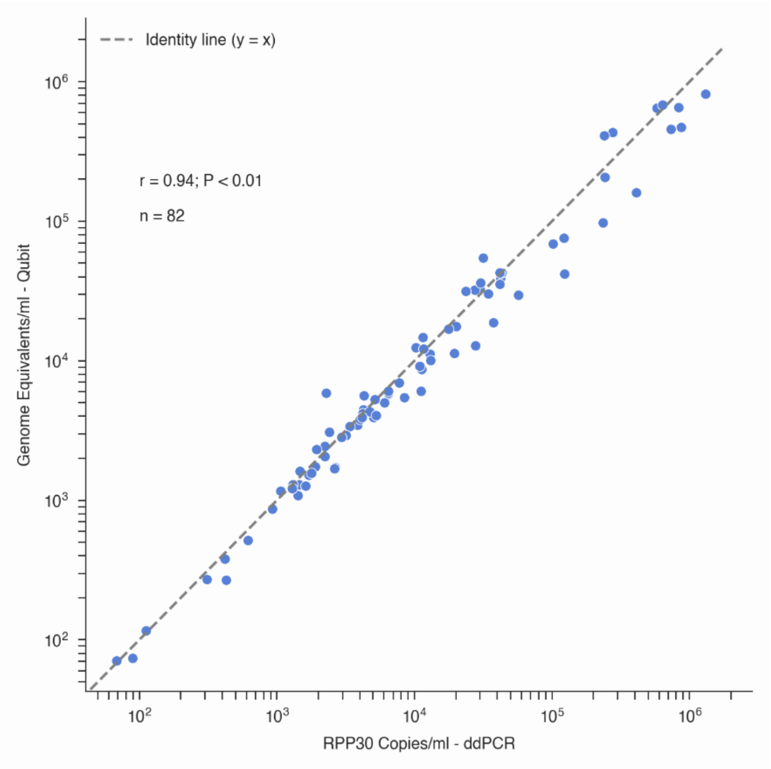

Figure S17. Agreement between Qubit- and ddPCR-based total cfDNA quantification, related to STAR Methods.
Scatterplot of plasma cfDNA concentrations (n = 82 samples) quantified with Qubit and ddPCR. *RPP30* gene copies were measured with ddPCR. Pearson's r correlation coefficient is shown. The grey line represents the identity line (y=x).
